# Ring contraction and ring expansion reactions in terpenoid biosynthesis and their application to total synthesis

**DOI:** 10.3762/bjoc.22.21

**Published:** 2026-02-17

**Authors:** Nicolas Kratena, Nicolas Heinzig, Peter Gärtner

**Affiliations:** 1 Institute of Applied Synthetic Chemistry, TU Wien, Getreidemarkt 9, A-1060, Vienna, Austria

**Keywords:** biosynthesis, rearrangement, ring contraction, ring expansion, skeletal editing, terpenoids, total synthesis

## Abstract

Terpenoids exhibit remarkable structural diversity, including highly complex ring-expanded or contracted carbocyclic skeletons. This review aims to explore intriguing examples of such ring-size alterations in all aspects of terpenoid synthesis. The current state-of-the-art regarding proposed biosynthetic pathways for terpenoids with unusual carbon skeleta, occurring either during initial cyclisation or subsequent oxidative tailoring, will be examined and discussed. Where possible, biogenetic relationships of closely related families of natural products will be contextualised by showing the mechanistic rationale for their interconversion. In the second part of this article, the application of bioinspired ring contraction and ring-expansion strategies in relevant natural product syntheses will be presented, demonstrating how synthetic chemistry can help to elucidate plausible biogenetic routes for structurally complex natural products.

## Introduction

The vast richness of structural diversity in terpenoid natural products has fascinated organic chemists for more than a century now [1–5]. With more sophisticated techniques for isolation and characterisation emerging over the decades, thousands of closely related compounds could be identified. Among these, the more structurally interesting, rearranged or highly oxidised members can often only be vaguely traced back to their biogenetic origin based on co-isolation and biochemical intuition. Synthetic approaches can sometimes help to solve these puzzling questions or even result in reassignment of the molecular structure [6–10]. Nevertheless, terpenoids with novel or highly uncommon carbon skeleta continue to attract interest from both biologists and chemists, as they often possess interesting biological properties owing to their unique ring systems, high degree of 3-dimensionality in their structures and oxidation patterns. As obtaining a detailed understanding of a biosynthetic pathway is a dauntingly complex and very labour-intensive process, knowledge about the precise origin of most rearranged terpenoids cannot be secured. Organic synthetic chemistry can help to fill gaps or evaluate, support or revise initially implausible proposed biogenesis routes by attempting to mimic these transformations (= bioinspired or biomimetic synthesis) [11–18]. In general, the biogenesis of unique carbon skeleton terpenoids can be broken down to several stages. First, a linear polyolefin precursor containing multiples of C_5_ will be assembled. In general, the cyclisation precursor will interact with a cyclase or synthase enzyme and form a “primary reactive species” which undergoes programmed termination to deliver the most common terpene frameworks. In some instances, these enzymes can also effect ring contraction or expansion directly during the initial cyclisation mechanism [19–27]. In many cases though, the ring-altering reaction instead takes place at a later stage of biosynthesis, when the oxidation state of the terpenoids is being adjusted [28–36]. Starting with an already substantial number of these common polycyclic frameworks and adding the almost unlimited variability for oxidative enzymatic C–H functionalisation, it is not surprising that terpenoids with completely unique carbon connectivity and ring systems are still discovered every single year.

In this review article, intriguing examples of apparent ring contraction or expansion reactions of carbocycles in terpenoids, both in biosynthesis and application of similar tactics in the total synthesis, are gathered. Our definition includes both radical and polar ring-size altering reactions, transannular cyclisations of macrocycles and cation-mediated rearrangements where fitting. Goal of this review is to gather and compare mechanistic proposals for the biogenesis of ring-size-altered terpenoids and highlight the utility of strategically including such a step in a natural product synthesis.

**Scheme 1:**
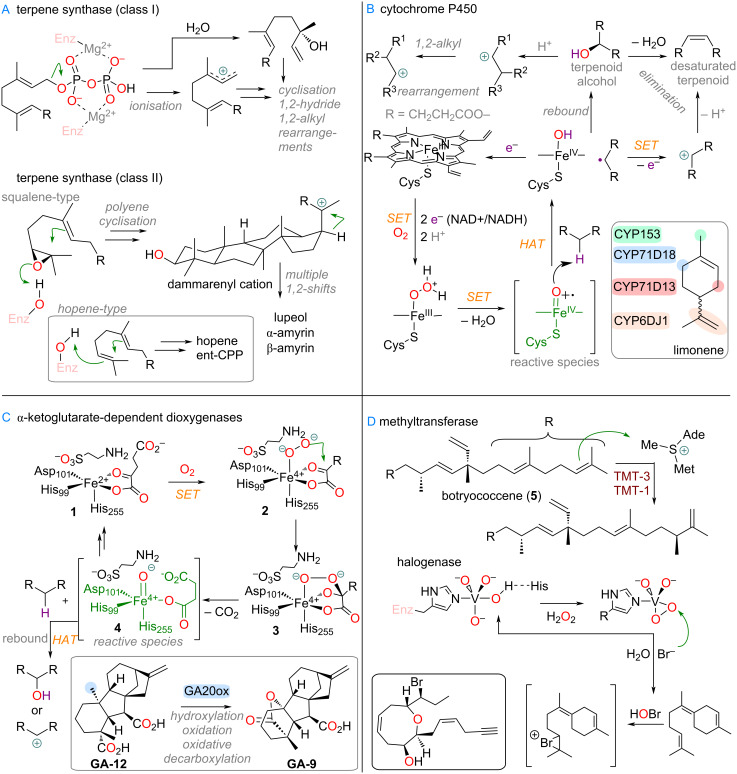
Mechanistic overview of enzymes involved in ring-size-altering reactions: A: Difference in ionisation mechanism in terpene synthases; B: General mechanism and summarised catalytic cycle of CYP450 oxidations; C: Summarised catalytic cycle of α-KG dependent dioxygenases, example of gibberellin acid biosynthesis; D: Importance of methyltransferases and halogenases in terpene biosynthesis, example of methylation of botryococcene (**5**).

To start, an overview of the different classes of enzymes, and thus common mechanisms, of ring-size-altering reactions for terpenes will be presented. In Scheme 1 the four most important manifolds for terpenoid modification in nature are depicted.

Terpene synthase enzymes are centrally important for determining the carbon skeleton and thus family the terpenoid belongs to. These cyclisation enzymes are divided into Class I (active site cleft contains multiple Mg^2+^-binding motifs which are responsible for the activation of the diphosphate group and ionisation) [25,37] and Class II (ionisation by olefin or epoxide protonation via a carboxylic acid in the active site) [26,38–39] and their reactions thus are mediated by carbocations and olefins, through careful preorganisation of the linear substrate. Alkyl and hydride shifts, stepwise or dyotropic rearrangements have been described to occur during terpene cyclisation, including multiple ring-size modifications.

The CYP450s play a central role for the oxidative functionalisation of terpenes, enabling the rich diversity of secondary metabolites [40–41]. They contain an iron-protoporphyrin core with a cysteine ligand [42] in the axial position, which cycles through different oxidation states (Fe^3+^/Fe^2+^/Fe^4+^) to form highly reactive oxo species which are effective at abstracting closely positioned aliphatic hydrogens via HAT (hydrogen atom transfer) [43]. The resulting alkyl radicals are often rebound to yield terpene alcohols [44–46] or epoxides [47], but other follow-up reactions are possible.

Next, the class of α-KG (ketoglutarate)-dependent dioxygenases also plays an important role, especially for terpenoids, and is therefore included [48–49]. These enzymes exhibit distinctly different Fe-containing active sites, typically consisting of two histidines, a carboxylate ligand (e.g., aspartate) and, of course, the ketoglutarate which is bound both via the C-2 ketone and C-1 carboxylate to form **1** (Scheme 1C). Upon its coordination with oxygen via **2** the ketal structure **3** can decarboxylate [50], generating another high-energy ferryl-oxo species **4** (bound with succinate) which can lead to hydroxylation or carbocation chemistry [51].

Finally, other classes of tailoring enzymes can also act as electrophiles and lead to ring-size modifications via carbocation formation and 1,2-shifts or are otherwise important in their biosynthesis (cf. below for botryococcene (**5**), Scheme 1D). Methyltransferases, which contain a closely bound *S*-adenosylmethionine (SAM) as methylating agent in the active site [52–53], and halogenases (e.g., vanadium-catalysed HOX synthesis, see Scheme 1D) [54–55] are of importance in that regard. Apart from these reactions, spontaneous non-enzymatic reactions can also be responsible for triggering ring-size change in terpenoids.

## Review

### Examples of biosynthetic ring-size adjustments

#### Ring contractions and expansions of terpenoids with secured biosynthetic routes

**Scheme 2:**
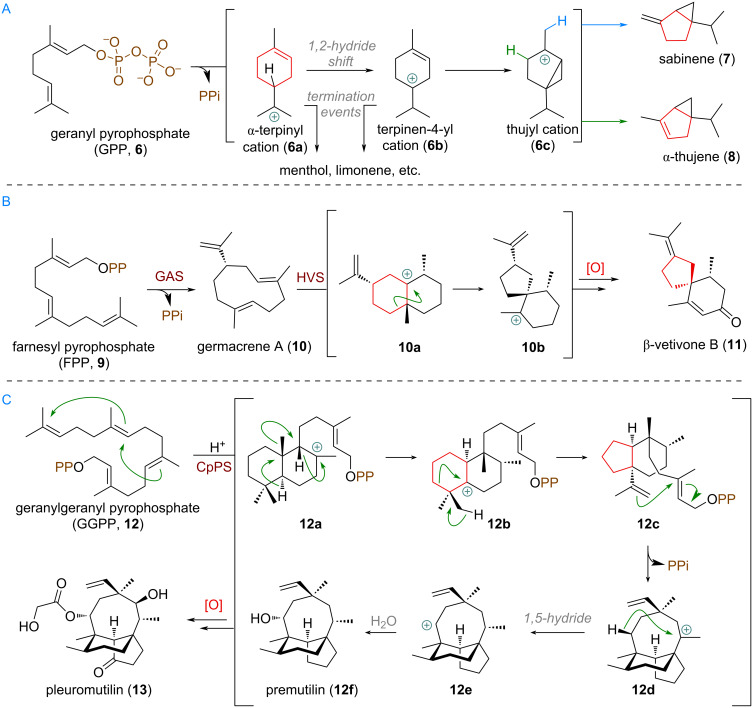
A: Ring contraction through involvement of carbocationic intermediates in thujane monoterpene biosynthesis; B: Spirocyclisation of a decalin system, initiated from germacrene A (**10**) during biosynthesis of the sesquiterpenoid β-vetivone B (**11**); C: Decalin-system ring contraction in pleuromutilin (**13**) biosynthesis through multiple 1,2-hydride and alkyl shifts.

The large family of C_10_-terpenes (monoterpenes) includes many linear compounds but also mono- and bicyclic systems. The variety of structures arises primarily through complex, enzymatically guided polyene cyclisation events of linear precursors, such as geranyl pyrophosphate (GPP, **6**). As the goal of this review is to cover mainly skeletal modifications arising from follow up biosynthetic reactions after cyclisation, only selected examples of ring-size altering fundamental steps in polyene cyclisation mechanisms will be presented now. One such example is the formation of the bicyclo[3.1.0]hexane system present in thujane monoterpenes [56] (see Scheme 2A). Starting from geranyl pyrophosphate (**6**), monoterpene cyclases first build up a 6-membered ring with an exocyclic carbocation, commonly referred to as α-terpinyl cation **6a**. From there, a 1,2-hydride shift gives rise to the isomeric terpin-4-yl cation **6b**. By way of cyclopropane formation, a different, so called, thujyl cation **6c** is conceivable. Elimination then furnishes the ring-contracted 5/3-ring systems from the original cyclohexyl intermediate to give sabinene (**7**) and α-thujene (**8**).

Another example of a 6→5 ring contraction can be found in the family of spirocyclic C_15_-sesquiterpenes, such as β-vetivone B (**11**, see Scheme 2B) [57–60]. The linear precursor farnesyl pyrophosphate (**9**) is first cyclised by germacrene A synthase (GAS) to its name-bearing product **10**. From here, protonation by vetispiradiene synthase (HVS) gives the eudesmane cation **10a**, which is a common intermediate in the biosynthesis of bicyclic sesquiterpenes, e.g., aristocholenes. In this particular enzymatic reaction, the 1,2-alkyl shift of a Wagner–Meerwein rearrangement is responsible for building up the spirocyclic carbon framework and ring contraction (**10b**). From here, follow up oxidations and olefin isomerisation afford β-vetivone B (**11**), a constituent of aromatic vetiver oil.

Finally, in the biosynthesis of the antibiotic pleuromutilin (**13**) [61–63] a similar ring contraction takes place (see Scheme 2C). Cyclisation is initiated via protonation of geranylgeranyl pyrophosphate (GGPP, **12**) at the terminal olefin with CpPS (= *Clitopilus passeckeranis* pleuromutilin synthase), forming the decalin system **12a**. A cascade of 1,2-shifts delivers the carbocation **12b** which undergoes ring contraction and elimination to give **12c**. From here, a second cyclisation towards **12d**, 1,5-hydride shift to secondary carbocation **12e** and follow-up oxidative decoration of **12f** affords the pleuromutilin molecule **13**.

**Scheme 3:**
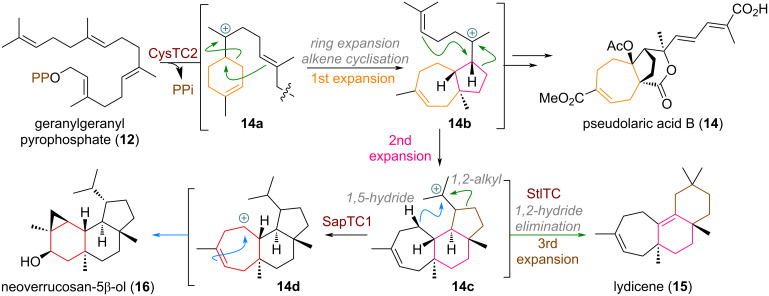
Examples of concerted ring expansions of carbocation intermediates in PxaTPS8-catalysed cyclisations towards fungal diterpenoids in nature and transannular cyclopropyl cyclisation.

A more recently unveiled example of a specific cyclase enzyme effecting a change in ring size during polyene cyclisation was found in pseudolaric acid B (**14**) biosynthesis [64–65]. In this case, the linear precursor **12** is first cyclised to give intermediate **14a** akin to the α-terpinyl cation (see Scheme 3). From here, quantum chemical calculations indicate that the subsequent 1,2-alkyl shift and olefin cyclisation occur in a single, concerted step. This concerted mechanism allows the reaction to bypass a high-energy, non-stabilised secondary carbocation intermediate that would result from a stepwise migration. This pathway is facilitated by the enzyme's active site, where aromatic residues (e.g., Tyr564) are proposed to provide the essential carbocation stabilisation (likely via cation–π interactions) and thus facilitate alkyl migration. The resulting 5,7-bicyclic carbocation **14b** is further elaborated and decorated by selective oxidation reactions to build up the family of pseudolaric acids. For example, after a second ring expansion, and additional 5-ring cyclisation by the pendant alkene a new tricyclic carbocation **14c** is formed. From here a 1,2-alkyl shift delivers the 7,6,6-ring system of lydicene (**15**) [66]. Alternatively, a 1,5-hydride shift affords the secondary carbocation **14d** which undergoes ring contraction to afford the cyclopropyl-containing natural product neoverrucosanol (**16**) [67–68].

**Scheme 4:**
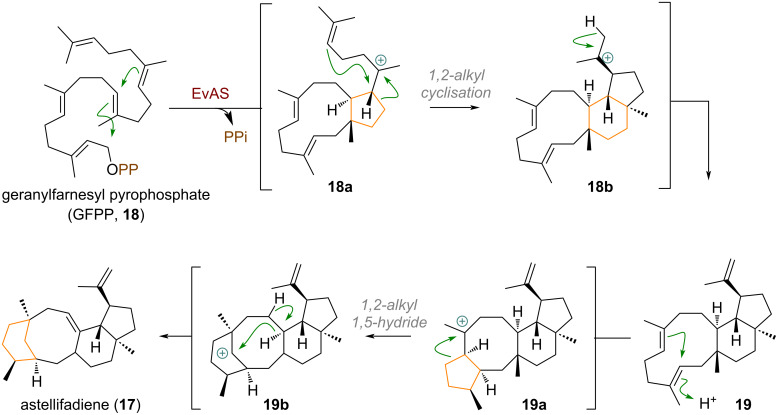
Sequential ring expansions during astellifadiene (**17**) synthesis reported by Abe and co-workers.

Another closely examined example of a similar ring expansion was documented by Abe et al. (see Scheme 4) to occur during the biosynthesis of astellifadiene (**17**), catalysed by the cyclase EvAS (= *Emericella variecolor* astellifadiene synthase) [69]. Geranylfarnesyl pyrophosphate (**18**) is first cyclised through 1,11- and 10,14-connections to the tertiary carbocation **18a**, and undergoes the previously described ring expansion, cyclopentane cyclisation (analogous to Scheme 3, see above) to **18b**. Elimination affords the triene intermediate **19**, followed, once again, by transannular cyclisation to give the tertiary carbocation **19a**. A subsequent second ring expansion affords the bridged system cation **19b**, which is quenched by a 1,5-hydride shift to finally provide astellifadiene (**17**).

**Scheme 5:**
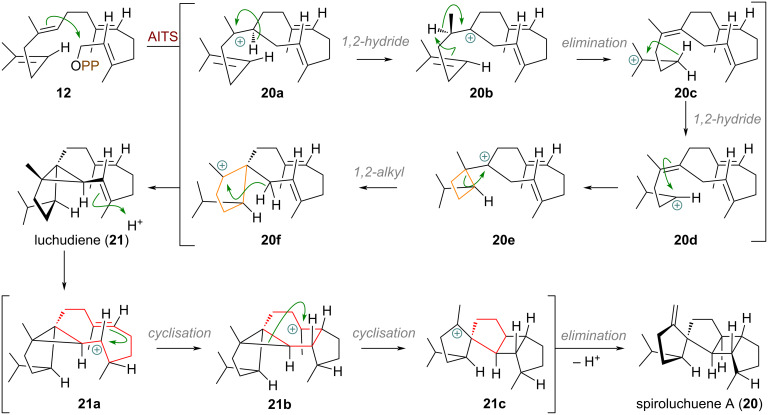
Cyclobutane ring expansion and sequential ring contractions catalysed by the synthase AITS in the biosynthesis of spiroluchuene A (**20**).

A distinct and intricate cyclisation cascade was elucidated by Dickschat and co-workers in 2023, describing the biosynthesis of the spirocyclic diterpene spiroluchuene A (**20**, see Scheme 5), catalysed by the synthase AlTS (*Aspergillus luchuensis* terpene synthase) [70]. Geranylgeranyl diphosphate (GGPP, **12**) is initially cyclised through the well-known 1,10-closure to the cation **20a**, followed by a sequence of hydride (**20b**) and proton shifts to cation **20c**, and a second hydride shift to **20d**. Subsequent cyclobutane formation via **20e** and a ring expansion furnishes cation **20f**, which is quenched by deprotonation with cyclopropanation to afford the key neutral intermediate luchudiene (**21**). One of the olefins in **21** is then reactivated by re-protonation, initiating a second cyclisation sequence that proceeds via cations **21a** and **21b**. Finally, a remarkable sacrificial carbocyclisation (rupture of the cyclopropane ring) at an aliphatic centre then generates cation **21c**, which is quenched by deprotonation to yield spiroluchuene A (**20**).

**Scheme 6:**
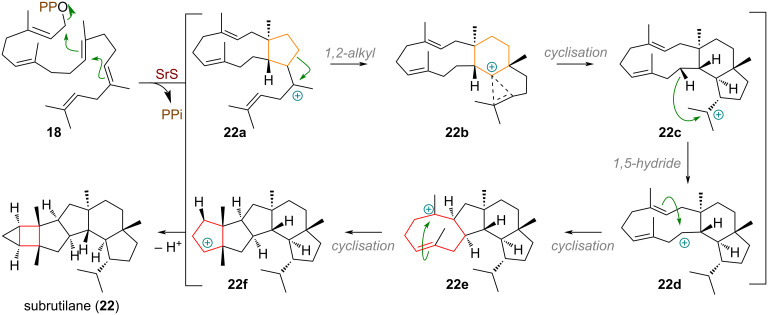
Ring expansion and transannular ring contraction of a cyclopentane to cyclobutane in the biosynthesis of the sesterterpene subrutilane (**22**) catalysed by SrS.

An illustrative example of a complex cyclisation cascade with multiple ring-size modifications was documented by Dickschat et al. during the biosynthesis of the saturated sesterterpene subrutilane (**22**, see Scheme 6), catalysed by the cyclase SrS (= *Streptomyces subrutilus* synthase) [71]. Geranylfarnesyl diphosphate (**18**) is first cyclised through 1,11- and 10,14-connections to the tertiary carbocation **22a**, which undergoes ring expansion (**22b**) and a 14,18-cyclisation to **22c**. A 1,5-hydride shift from the macrocycle generates cation **22d**. Subsequent 2,9- and 3,7-cyclisations afford the key carbocation intermediate **22f**, which is finally quenched by a deprotonation with concurrent cyclopropanation, effectively contracting a cyclopentane to a cyclobutane, to yield subrutilane (**22**).

**Scheme 7:**
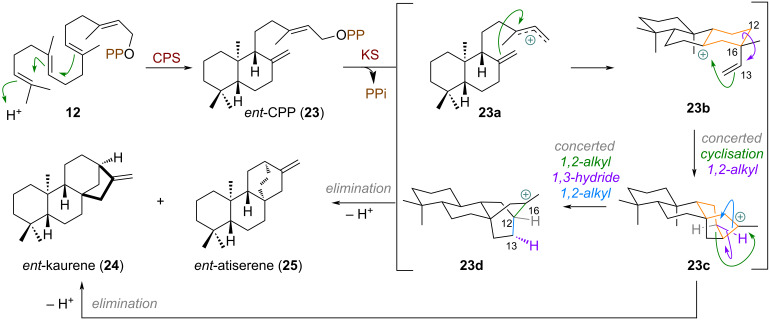
Computationally elucidated concerted cyclisations/alkyl/hydride shifts during the biosynthesis of the terpenes *ent*-atiserene (**25**) and *ent*-kaurene (**24**).

Based on quantum chemical studies by Tantillo and Hong the biosynthesis of kaurene diterpenes includes interesting, concerted alkyl migration steps [72–73]. Starting from GGPP (**12**) the class II terpene synthase CPS (= *ent*-Copalyl diphosphate synthase) catalyses decalin formation through cationic polyene cyclisation of *ent*-copalyl diphosphate (**23**) (Scheme 7). From here, different enzymes (e.g., *ent*-Kaurene synthase) can effect cyclisation of cation **23a** to the pimarenyl cation **23b**, from which earlier works [74–75] proposed a stepwise cyclisation and alkyl shift to occur. The secondary carbocations which are invoked in this process were found to not be the likely operational intermediates, as calculations showed instead a concerted rearrangement towards the tertiary cation **23c** to be more likely. From here, *ent*-kaurene (**24**) is obtained directly after elimination. The formation of *ent*-atiserene (**25**) involves a more dramatic rearrangement to reach the tertiary carbocation **23d**. A triple asynchronous shift occurs, consisting of C12-alkyl (13→16), 1,3-hydride (12→13), and C-13 alkyl shift (16→12). This complex, concerted process directly converts the tertiary cation **23c** into the tertiary cation **23d** completely avoiding secondary cations and resulting in another 5→6 ring expansion to the bridged system of **25**.

**Scheme 8:**
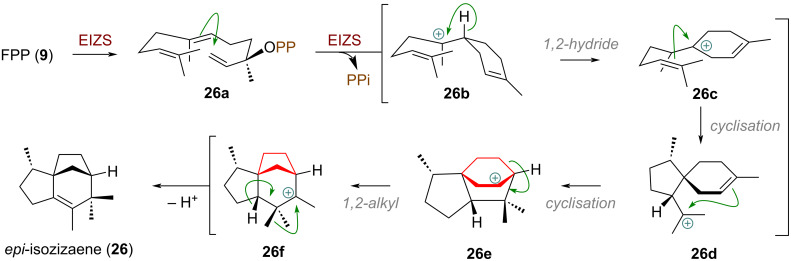
Cyclisation events and 6→5-ring contraction during the construction of *epi*-isozizaene (**26**) catalysed by EIZS.

An early example of a sesquiterpene cyclisation sequence with a ring contraction was documented by Cane et al. during the investigation of *epi*-isozizaene (**26**, Scheme 8), catalysed by the synthase EIZS (= *Epi*-isozizaene synthase) [76]. Farnesyl diphosphate (**9**) is first ionised and isomerised to (3*R*)-nerolidyl diphosphate (**26a**), which undergoes cyclisation to form the bisabolyl cation (**26b**). A subsequent 1,2-hydride shift yields cation **26c** which undergoes spirocyclisation to generate the acorenyl cation (**26d**). This key intermediate then undergoes a sequence of further cyclisation (**26e**) and crucial ring contraction via 1,2-alkyl shift to **26f**. Methyl migration and quenching by elimination finally afford *epi*-isozizaene (**26**).

**Scheme 9:**
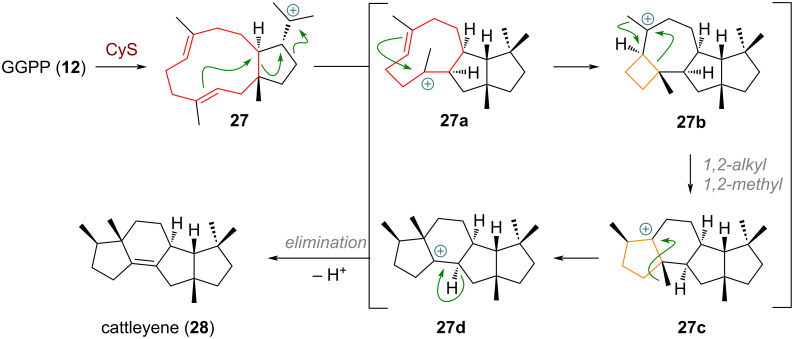
Transannular cyclisations and 4→5-membered ring expansion through dyotropic 1,2-rearrangement of alkyl and methyl groups in the biosynthesis of cattleyene (**28**).

Investigative efforts into actinomycetes biosynthesis by Dickschat et al. through deuterium labelling revealed another example for a 4→5 ring expansion which is depicted in Scheme 9 [77]. Intermediate **27** is obtained like shown before (see above, Scheme 4 and Scheme 6), through the enzyme cattleyene synthase (CyS). Next, a concerted ring expansion/ring contraction and additional 2,10-cyclisation delivers the macrocyclic cation **27a**. A further 3,6-cyclisation forms the cyclobutane intermediate **27b** which can undergo ring expansion via dyotropic rearrangement to give the tetracyclic system of **27c**, followed by 1,2-methyl migration (**27d**) and elimination to furnish cattleyene (**28**).

**Scheme 10:**
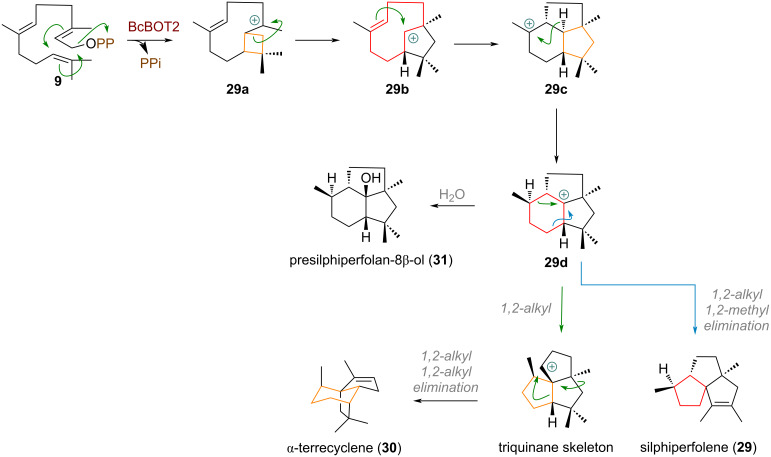
Ring expansion in presilphiperfolan-8b-ol (**31**) biosynthesis and ring contraction of the presilphiperfolan-8-yl cation (**29d**) towards different sesquiterpene skeleta.

During the investigation of botrydial biosynthesis the groups of Collado, Cane and Viaud could identify a protein titled BcBOT2 (= Botrytiscinerea BOTrydial) with similarity to microbial terpene synthases [78]. Starting from farnesyl pyrophosphate (**9**) BcBOT2 catalyses a rare, formal [2 + 2] cycloaddition to form the cyclobutyl carbenium ion **29a** which undergoes spontaneous ring expansion via 1,2-alkyl shift (see Scheme 10). The secondary carbocation on the 5-membered ring in **29b** undergoes additional cyclisation (and ring contraction from a 9-membered ring to a 6/5 bicycle) to give tertiary cation **29c**. From here a 1,2-hydride shift leads to intermediate **29d** (presilphiperfolan-8-yl cation), a precursor which was invoked to be involved in the biosynthesis of various sesquiterpenes. Two noteworthy examples are given here: either of the two 1,2-alkyl shifts of the cyclohexane accomplishes ring contraction. Following the blue arrow in the structure of **29d** (Scheme 10) a second 1,2-methyl migration is required to furnish silphiperfolene (**29**) [79]. If instead, the bond indicated with the green arrow migrates the triquinane skeleton is assembled. Notably, Yan reported recently [80] that the sesquiterpene α-terrecyclene (**30**) is formed through the same carbocation **29d**, according to the mechanistic proposal by Coates [81]. By interception of the cation with water we arrive at presilphiperfolan-8β-ol (**31**)

**Scheme 11:**
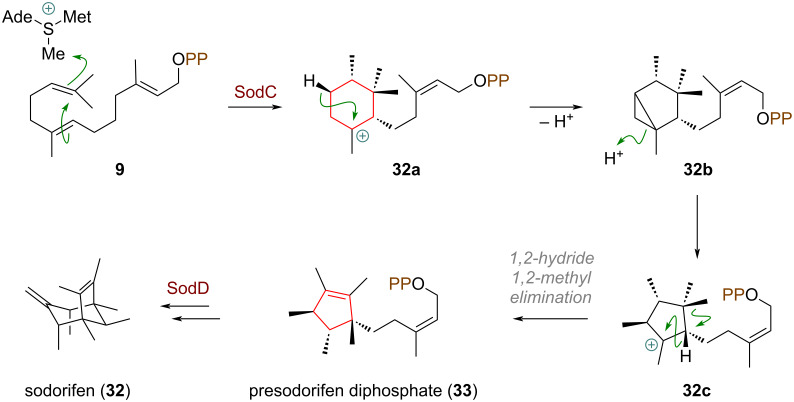
Ring contraction via transannular cyclopropanation and opening of cyclopropane in the biosynthesis of the microbial terpene sodorifen (**32**).

Dickschat and co-workers reported an example for a ring contraction during the biosynthesis of sodorifen (**32**) [82]. The cyclisation is triggered by the C-methyltransferase SodC (= pre-sodorifen synthase) which catalyses methyl cation transfer from SAM towards the terminal olefin in **9**, a rare event in terpene cyclisation chemistry, resulting in 6,11-ring closure (**32a**, see Scheme 11). From here, a dyotropic rearrangement can give cation **32c** directly, alternatively the mechanism could be enabled by deprotonation through an active-site base (**32b**) and re-protonation of the cyclopropane moiety (**32c**). A 1,2-hydride shift, followed by a 1,2-methyl shift towards that same carbon and an elimination, forms the tetrasubstituted double bond in **33**, the precursor for sodorifen (**32**), which is furnished by the class I synthase SodD.

**Scheme 12:**
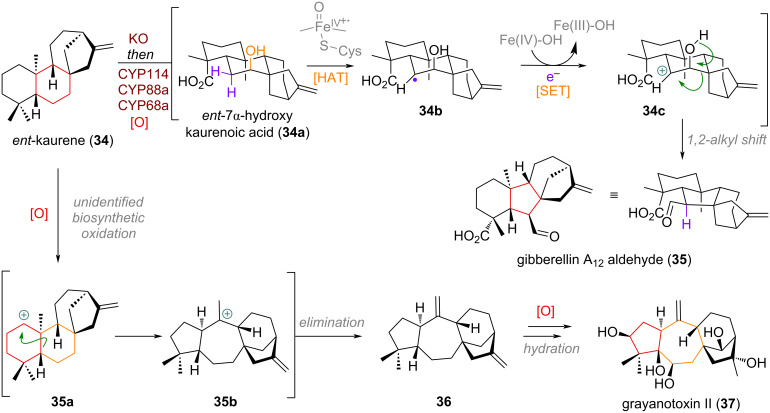
The crucial CYP450-catalysed oxidative rearrangement defining the skeleton in gibberellin biosynthesis and selective C-1 oxidation and ring expansion/contraction towards grayanotoxin II (**37**).

A well-studied example of an oxidative 6→5-membered ring contraction in terpenoid biosynthesis can be found in the formation of the gibberellin family [83–84]. In this instance, the terpene *ent*-kaurene (**34**, see Scheme 12) is being oxidised both at one of the methyl groups residing at C-4 and the C-7 methylene to *ent*-7α-hydroxykaurenoic acid (**34a**). The hydroxy group in **34a** can further engage with a CYP450 enzyme at C-6 (different CYP isoforms responsible in different genii) to form an alkyl radical **34b** which upon further SET forms an intermediate carbocation **34c**, which collapses under an 1,2-alkyl shift to reveal the key intermediate in gibberellin synthesis, gibberellin A_12_ aldehyde (**35**). Alternatively, the *ent*-kaurene core is known to be likely oxidised at C-1 [85] by a thus far unknown biosynthetic oxidation to deliver a secondary carbocation **35a**, which can undergo tandem ring expansion/contraction from the 6,6 to the 7,5 system (**35b**). After elimination the precursor **36** to the large family of grayanotoxin natural products is reached (grayanotoxin II (**37**) is depicted exemplarily).

**Scheme 13:**
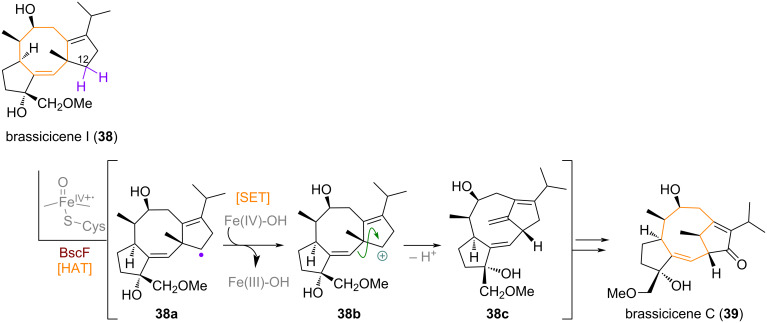
CYP450-mediated oxidation of cyclopentane methylene expanding the 8-membered ring in the biosynthesis of brassicicene natural products.

Another well-studied example of a CYP450-catalysed oxidation triggering a change in ring size was found to occur during the biosynthesis of some brassicicene natural products such as brassicicene I (**38**) [86]. The 5-8-5 ring system present in these natural products (see Scheme 13) is transformed into a 5/9/5-bridged system through a methylene C-12–H oxidation mediated by the enzyme BscF, triggering a Wagner–Meerwein rearrangement after radical-polar crossover (**38a** to **38b**) and elimination at the bridgehead methyl, resulting in the *exo*-olefin. The final intermediate **38c** is further elaborated by enzymatic oxidation to give brassicicene C (**39**) from brassicicene I (**38**).

**Scheme 14:**
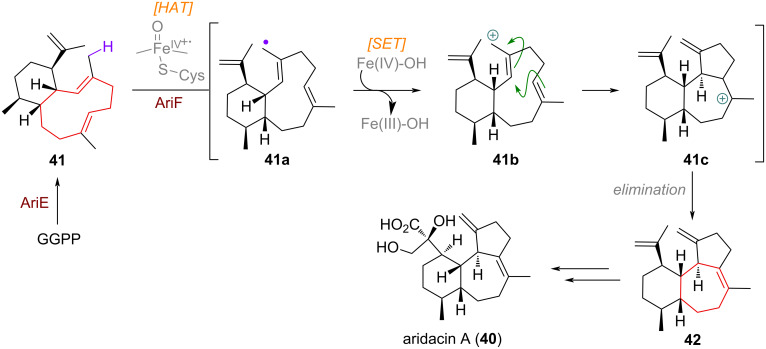
CYP450-mediated oxidation of an exocyclic methyl group to effect transannular cyclisation across the 10-membered macrocycle towards aridacin A (**40**).

In the biosynthesis of aridacin A (**40**), the initial 6,10-membered bicyclic product **41**, after AriE-catalysed polyene cyclisation engages in a HAT with the CYP450 enzyme AriF, abstracting a hydrogen from the C-20 methyl group on the macrocycle [87–88]. The resulting allylic radical **41a** can cyclise directly, subsequently giving a different tertiary radical which can get oxidised and quenched by elimination (not depicted in Scheme 14). Alternatively, a SET can occur directly on the methyl radical, delivering allylic carbocation **41b**, which cyclises barrierless to give tertiary carbocation **41c**. Regioselective, endocyclic elimination delivers the unoxidised terpene precursor **42** towards aridacins.

**Scheme 15:**
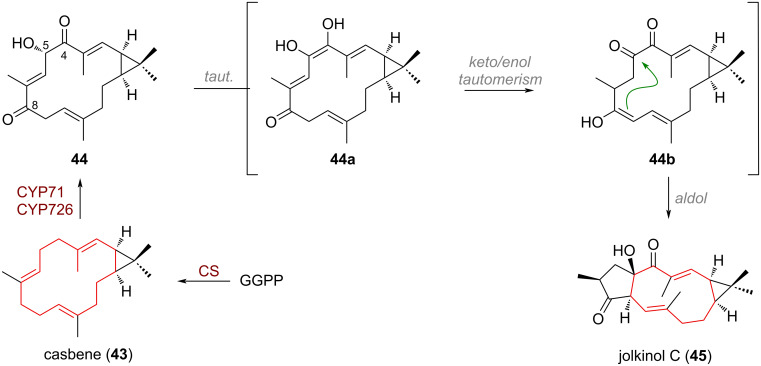
Non-enzymatic transannular aldol reaction enables the formation of the 5/13/3-tricyclic ring system present in lathyrane diterpenoids from the casbane skeleton.

In the rich family of *Euphorbia* diterpenoids various ring systems are conjectured to be biosynthetically related, such as the casbane, lathyrane, tigliane, and ingenane skeletal [89–90]. The transannular aldol reaction of an oxidised casbene (**43**, produced from GGPP by casbene synthase, CS) product **44** has been studied closely by the groups of Graham and Hamberger who investigated the BGC present in *Jatropha curcas* and *Euphorbia lathyris,* respectively (see Scheme 15) [91–93]. They found that two CYP450 enzymes, CYP71 and CYP726 were primarily responsible for C-4, C-5 and C-8 oxidation of casbene **43** to form intermediate **44** which can isomerise by means of keto–enol tautomerism to give **44a**. An additional keto–enol tautomerism allows for the transient formation of **44b**, a new enol engaging in an aldol addition towards the highly electrophilic α-diketone moiety. With this, the bicyclic lathyrane skeleton of jolkinol C (**45**) is assembled selectively from casbene.

**Scheme 16:**
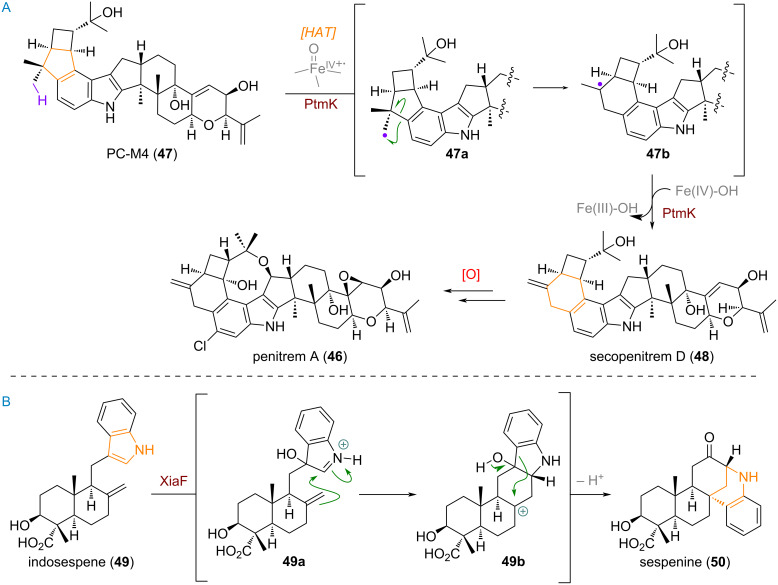
A: Oxidative ring expansion of a cyclopentane by incorporation of a methyl group in the biosynthesis of penitrem A (**46**); B: Ring expansion and rearrangement of the indole moiety in the biosynthesis of sespenine (**50**).

The large family of indole meroterpenoids [94–98] also contains an interesting, enzyme-mediated ring-expansion reaction. According to the detailed research of their biosynthesis carried out by Oikawa et al. [99–100] the terpenoid penitrem A (**46**) is formed from PC-M4 (**47**, see Scheme 16A). This precursor, exhibiting a 5-membered ring annulated onto the indole core, reacts in a HAT reaction under mediation of PtmK, an enzyme possessing an iron(IV)–oxo metal centre, to deliver the methyl-centred radical **47a**. From here, the 5→6 ring expansion takes place via radical mechanism giving rise to the stabilised tertiary radical **47b**. Oxidation of this radical to the corresponding carbocation and elimination leads to secopenitrem D (**48**) directly. From here another oxy-cyclisation and aromatic chlorination leads to the most complex member of the indole meroterpenoids, penitrem A (**46**).

Another example of a ring expansion reaction, in this case of the aromatic 5-membered ring of an indole, was discovered to be operational in the biosynthetic pathway towards xiamycins and dixiamycins (see Scheme 16B) [28,101–105]. The rearrangements commence by oxidation of C-3 in the indole ring of indosespene (**49**) by the enzyme XiaF, giving rise to iminium intermediate **49a**. Attack of this iminium by the pendant exocyclic olefin affords carbocation **49b**, which is quenched by a 1,4-alkyl shift of the aromatic system, and establishment of a C–O double bond at C-12 to give sespenine (**50**) as the product.

**Scheme 17:**
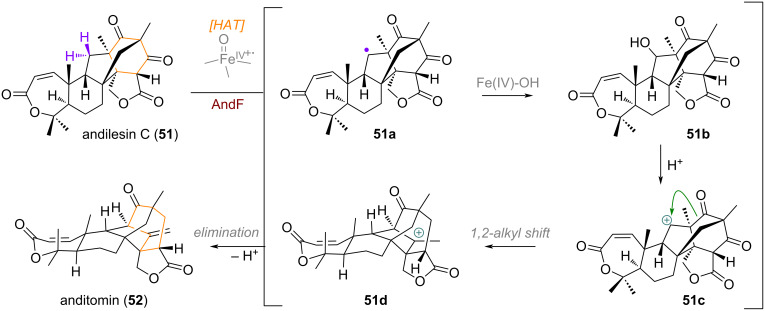
Rearrangement and ring expansion in the construction of the complex bridged carbon framework of anditomin (**52**), mediated by AndF.

In meroterpenoid biosynthesis, the transformation of andilesin C (**51**) towards the anditomin skeleton, as investigated by Abe and co-workers, is also an interesting example for ring expansion [106–108]. The PhyH-like dioxygenase AndF abstracts a hydrogen from C-11 of **51** giving rise to secondary alkyl radical **51a** (see Scheme 17). An iron(IV)-hydroxy-mediated rebound then results in the hydroxylation of this position. Upon E1 elimination of alcohol **51b**, carbocation **51c** is generated and undergoes a 1,2-alkyl shift of the ketone moiety, forming a new bridged 7-membered ring (**51d**), and completing the skeletal adjustment. A final elimination to the exocyclic olefin delivers the complex, caged structure of anditomin (**52**).

**Scheme 18:**
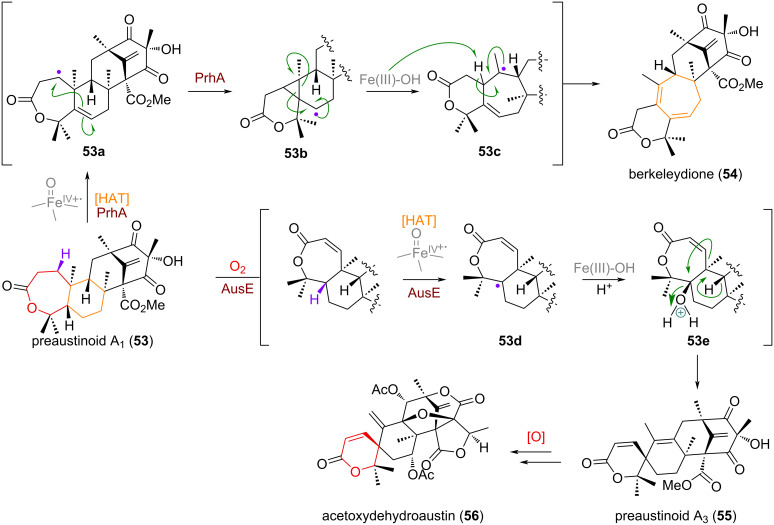
Ketoglutarate-mediated oxidations of preaustinoid A_1_ (**53**) en route to complex meroterpenoids, B-ring expansion via radical skeleton re-shuffling, A-ring contraction via Wagner–Meerwein spirocyclisation.

Apart from this D-ring expansion of 3,5-dimethylorsellinic acid (DMOA)-derived meroterpenoids, Abe and co-workers were also able to show enzymatically controlled pathways for the B-ring expansion and A-ring spirocyclisation and contraction [109]. From the common precursor preaustinoid A_1_ (**53**) a HAT step mediated by the iron(IV)-oxo enzyme PrhA generates a radical (**53a**) at C-1 of the terpenoid framework (see Scheme 18), resulting in cyclopropanation to give radical **53b**. Subsequent cyclopropane opening leads to the ring-expanded B-ring centred, tertiary radical **53c**. Finally, abstraction of the allylic hydrogen in this product by an iron(III)-species affords the 6/7/6/6 bridged framework of berkeleydione (**54**). A different iron(IV)-oxo enzyme AusE is also capable of selectively abstracting the 5α-hydrogen atom, giving rise to alkyl radical **53d**, which gets hydroxylated via rebound to give intermediate **53e** [110]. Carbocation formation at C-5, followed by a 1,2-alkyl shift and elimination thus furnishes the sister natural product preaustinoid A_3_ (**55**), thought to be the precursor to the highly oxidised meroterpenoid acetoxydehydroaustin (**56**).

#### Other interesting, proposed ring-size changing reactions in terpenoid biosynthesis

**Scheme 19:**
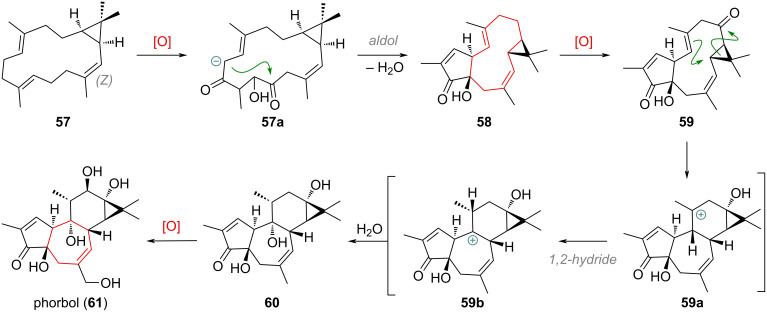
Proposed putative biosynthetic formation of the tigliane skeleton from an *E*,*E*,*Z*-triene.

Moving on from these carefully characterised biosynthetic transformations, where often single steps were carried out with specific enzymes or isotope-labelled substrates to prove origin, we now move to the much larger body of speculative biosynthetic relationships. From macrocyclic lathyrane frameworks such as jolkinol C (**45**), the formation of complex polycyclic tiglianes and ingenanes is also invoked, even though detailed studies have still not been performed [111–112]. The required C-8→C-14 cyclisation would require a C-nucleophile to be present at C-14 (next to the cyclopropyl group), attacking the C-8 ketone (e.g., in **45**). While the topological relation of lathyranes and tiglianes is immediately apparent, the mechanism and required pre-functionalisation for the 8-14 cyclisation are not trivial and have thus far not been supported by extensive synthetic model studies [113–114]. Instead, an alternative biosynthetic pathway towards tiglianes from a partly *Z*-configured casbene precursor **57** was proposed and is depicted in Scheme 19 [115–116]. Crucially after the formation of the 5/13/3-tricyclic system in **58** as before, an oxidation next to the cyclopropane would allow 1,2-migration of the cyclopropyl system after nucleophilic attack by one of the olefins in **59**. After a 1,2-hydride shift (**59a** to **59b**) and nucleophilic capture of the tertiary cation to give **60**, the full skeleton of tiglianes (e.g., phorbol (**61**)) would be assembled.

**Scheme 20:**
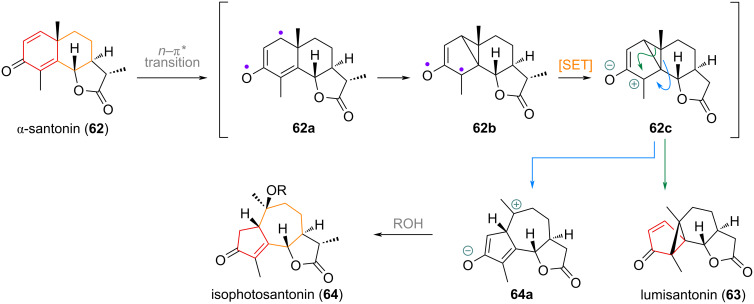
Photocatalytic tandem ring expansion/contraction of santonin to give photosantonin products and guaiane carbon skeleta.

An early example of a puzzling ring contraction observed in natural product chemistry was the transformation of santonin (**62**, see Scheme 20) into the ring-contracted 5-membered compounds lumisantonin (**63**) and the 5,7-membered ring-expanded guaiane system **64** [117–119]. The mechanism for this photochemical transformation involves transformation of **62** to a diradical at the unsaturated ketone (**62a**), cyclisation to form an intermediary highly substituted cyclopropane (**62b**) followed by single-electron transfer to the zwitterionic **62c** and rearrangement to either the dense connectivity of lumisantonin (**63**), or carbocation capture by solvolysis of intermediate **64a** to afford isophotosantonins **64**. The closely related guaiane natural products are not believed to be synthesised in nature via this mechanism and instead are formed by different termination events during polyene cyclisation of a key intermediate [120–121]. Due to the early discovery of santonin [122–124] in 1830 and the correct elucidation of its structure almost 100 years later, this example with negligible biosynthetic relevance is also included.

**Scheme 21:**
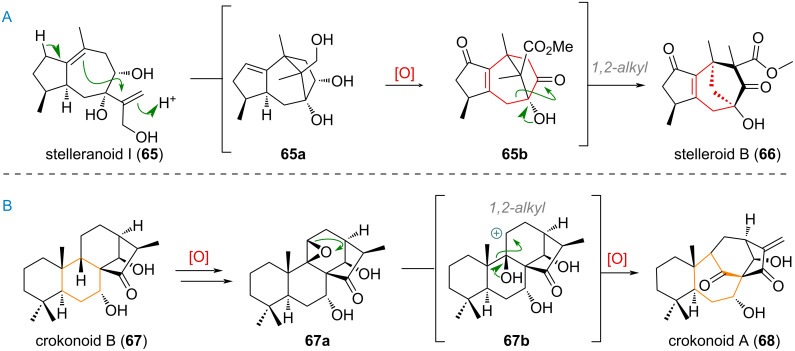
A: Proposed biosynthesis of stelleroid B (**66**) from stelleranoid I (**65**) by ketol rearrangement; B: oxidation and ring expansion in the biogenesis of crokonoid A (**68**) from crokonoid B (**67**).

The proposed biosynthesis of stelleroids [125–126] features an α-ketol rearrangement leading to a 7→6 ring contraction (see Scheme 21A). After a protonation-induced cyclisation of the bicyclic precursor **65** to the bridged system **65a**, and exhaustive oxidation to give intermediate **65b**, the structure of stelleroid B (**66**) is reached by a final 1,2-alkyl shift. The kaurene-derived product crokonoid A (**68**) was traced back to its co-isolated compound crokonoid B (**67**) by oxidation, carbocation formation and 1,2-alkyl shift in a semi-pinacol rearrangement [127]. The authors of the isolation report proposed an epoxide **67a** as initiating species, but a diol could also serve in this function (see Scheme 21B). Upon formation of the secondary carbocation **67b**, a 1,2-alkyl migration is invoked, giving rise to the bridged polycyclic system of **68**.

**Scheme 22:**
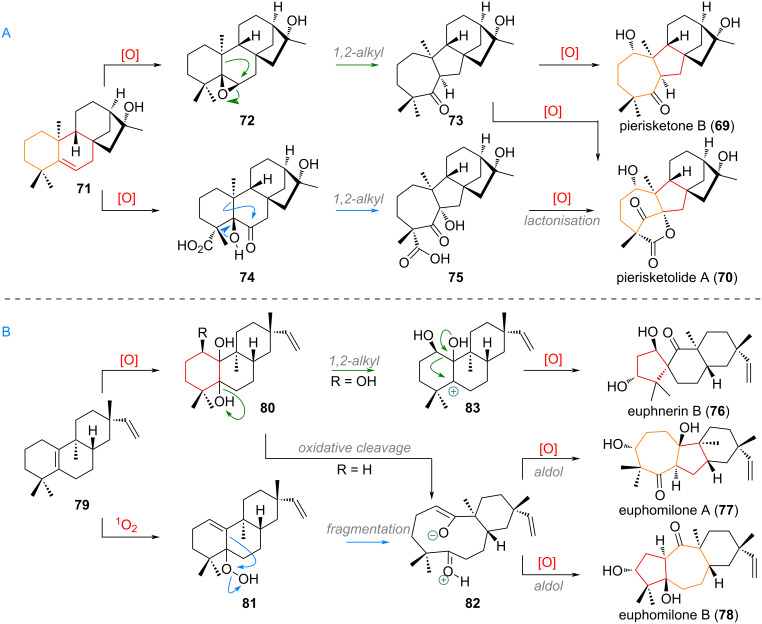
Singular examples of A,B-ring contractions and expansions in the biosynthesis of sesquiterpenoids euphomilones A (**77**) and B (**78**), euphnerin B (**76**), pierisketone B (**69**) and pierisketolide A (**70**).

Apart from these larger classes of sesquiterpenoids there are many singular examples of rearranged and ring-size-modified kaurene derivates, such as pierisketone B (**69**) and pierisketolide A (**70**). These 7,5,6,5-systems were proposed by the group responsible for their isolation [128] to be built up through oxidation of precursor **71** at C-5 and C-6 to give an epoxide **72**, which could undergo semi-pinacol rearrangement to the 7-membered ketone **73**. From here, a simple follow up oxidation could give both compounds (see Scheme 22A).

Alternatively, one can imagine an exhaustively oxidised intermediate like **74** undergoing α-ketol rearrangement to directly deliver the hydroxy group at C-6 (acid **75**) for intramolecular lactonisation towards pierisketolide A (**70**). In the biogenesis of euphnerin B (**76**) and euphomilones A (**77**) and B (**78**) the tricyclic precursor **79** was invoked (see Scheme 22B) [129]. This compound could undergo oxidation at the alkene (and C-1) to give intermediate **80**, which after oxidative cleavage affords the enolate of diketone **82** which can undergo transannular aldol addition to give either product **77** or **78** after proton exchange. The same intermediate diketone could be theoretically obtained directly from singlet-oxygen-mediated oxidation of the olefin through hydroperoxide **81** and Hock rearrangement [130–132]. Finally, euphnerin B (**76**) is furnished after 1,2-alkyl shift in **83** towards the C-5 carbocation, to afford the 5,6-spirocycle.

**Scheme 23:**
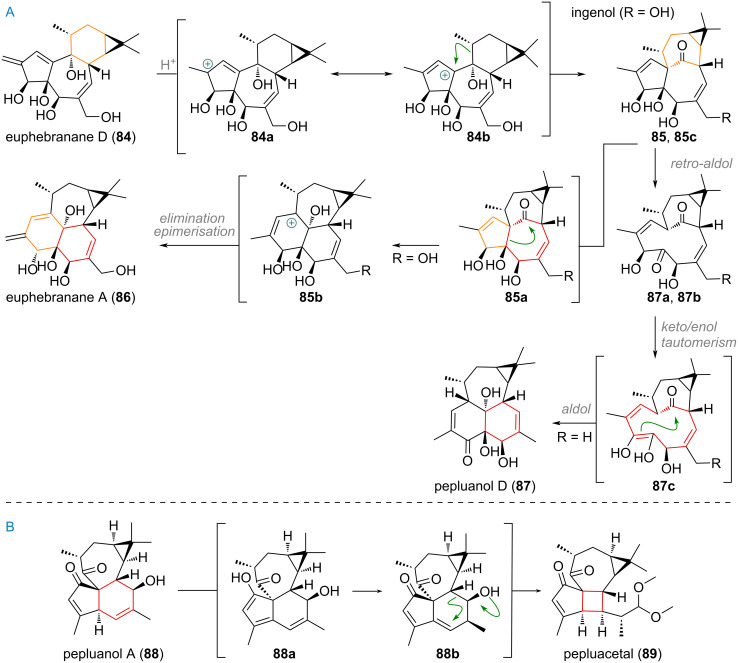
A: plausible proposed biosynthetic pathway for the tigliane/ingenane skeletal rearrangement and 1,2-shift ring contraction/expansion towards 6/6/7/3 diterpenoids euphebranane A (**86**) and pepluanol D (**87**); B: proposed biosynthetic pathway from pepluanol A (**88**) to give pepluacetal (**89**).

The transformation of these 5/7/6/3-membered systems into the ingenane skeleton was exploited to great effect in multiple independent total syntheses of these molecules (vide infra). It is conceivable that an analogous process, starting with oxidation at the methyl group to form an allylic carbocation, 1,2-hydride shift and finally semi-pinacol rearrangement is also responsible for the re-shuffling of the carbon skeleton in nature. While the connection of casbanes, lathyranes, tiglianes, ingenanes and even jatrophanes has been described and was the subject of previous studies, no detailed studies on the transformations connecting all these families have been performed thus far [133–136]. Novel *Euphorbia* diterpenoids recently isolated by Wang, Zheng and Yu in 2024 (see Scheme 23A) could be considered “missing links” offering an explanation as to how the strained inside-outside bridgehead of ingenol (**85**) is formed [137]. Precursor compounds such as euphebranane D (**84**) could, upon protonation at the exocyclic olefin give a stabilised carbocation **84a/84b**. This would allow for the 1,2-alkyl shift and ring expansion to take place, giving ingenol (**85**). From there, the related ring-expanded diterpenoid euphebranane A (**86**) was proposedly obtained by 1,2-alkyl shift of **85a** to give carbocation **85b** and finally **86** by elimination to the diene and epimerisation of the hydroxy group. An alternative mechanistic rationale for the expansion/contraction reaction from the 5/7/7/3-ring system towards a 6/6/7/3-system was proposed by Qiu, Zhou and Yue et al. in the isolation reports of pepluanols A–D [138–140]. Here, a retro-aldol reaction was invoked to cleave the C–C bond rupturing the 5- and 7-membered ring. The C-4 ketone can then enolise in a way that enables selective C-4 to C-14 ketone aldolisation furnishing pepluanol D (**87**). The same authors also proposed a putative biosynthetic origin for the intriguing 5/4/7/3-ring system of pepluacetal (**89**) from pepluanol A (**88**) by olefin isomerisation (**88a** to **88b**) and 1,6-conjugate addition to build up the new 4-membered ring (see Scheme 23B). Finally, acetal formation on the novel carbaldehyde furnished the structure of pepluacetal (**89**).

**Scheme 24:**
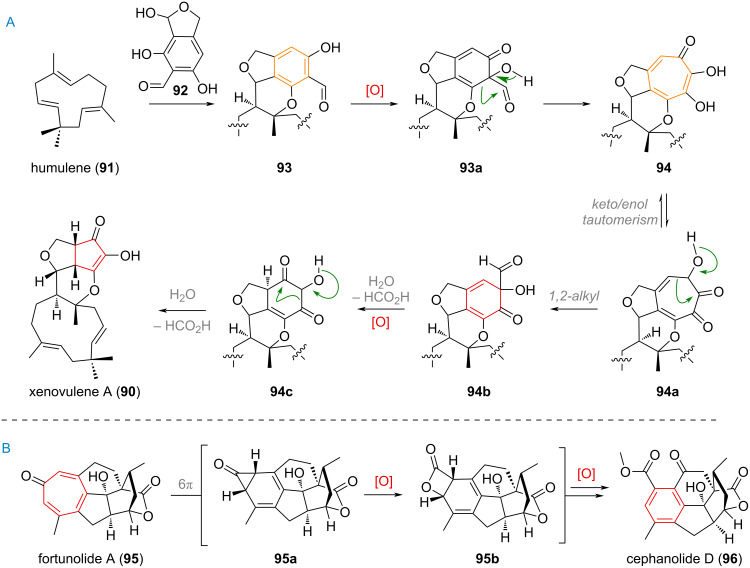
A: Multiple ring-size alterations during xenovulene A (**90**) biosynthesis; B: Ring contraction and rearomatisation in the biosynthesis of cephalotaxus diterpenoids.

An interesting example for both the ring expansion and contraction of benzene rings in nature was unveiled during investigations into the biosynthesis of xenovulene A (**90**, Scheme 24A) [141–143]. The terpenoid precursor **91** and the polyketide precursor **92** are merged to give rise to the tricyclic system of **93**. Oxidation of the electron-rich aromatic system in **93a** leads to a ring expansion to the tropolone system **94** via 1,2-shift. A similar ring contraction can now take place after keto/enol tautomerism to **94a** to deliver dearomatised intermediate **94b** which undergoes decarboxylation to give diketone **94c** after oxidation. After another 1,2-shift to the 5-membered ring a second formyl equivalent is removed to give the final product xenovulene A (**90**).

A different mechanism for the ring contraction of the tropolone system in *Cephalotaxus* diterpenoids was proposed and is depicted in Scheme 24B [144–145]. Starting from a precursor such as **95** it involves a 6π-electrocyclisation, resulting in a highly reactive, transient cyclopropyl ketone **95a** which undergoes Baeyer–Villiger oxidation to **95b** and finally rearomatisation and esterification to give cephanolide D (**96**) from fortunolide A (**95**).

**Scheme 25:**
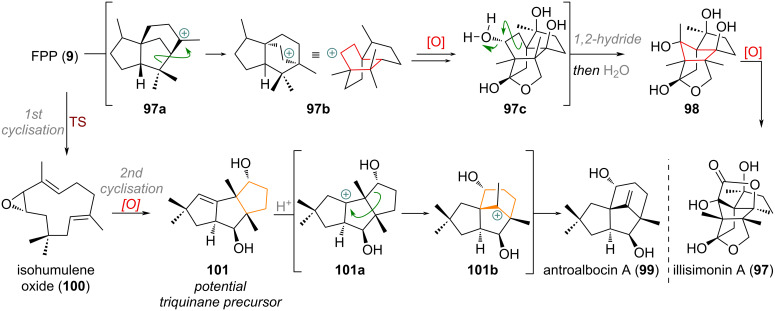
Proposed biosyntheses of the complex, polycyclic terpenoid illisimonin A (**97**) and the bridged antroalbocin A (**99**).

The complex sesquiterpenoid illisimonin A (**97**, see Scheme 25) was isolated in 2017 and traced back biosynthetically to FPP (**9**) and the cedrane cation **97a** [146]. From here a 1,2-alkyl shift and aqueous quench of the cation **97b**, expanding the 5-membered ring into the *allo*-cedrane carbon framework, furnishes the proposed biogenetic precursor **97c**. Following protonation of the bridgehead alcohol, a Wagner–Meerwein rearrangement, and a 1,2-hydride shift builds up the tricyclo[5.2.1.01,6]decane skeleton **98**.

The bridged sesquiterpenoid antroalbacin A (**99**) was isolated in 2018 and traced back to the biogenetic precursor isohumulene oxide (**100**, see Scheme 25) [147–148]. Cyclisation of **100** by an unknown enzyme builds up the triquinane skeleton, which can be oxidised to the precursor **101**. The ring enlargement is then proposedly initiated by protonation of the olefin giving rise to **101a**, 1,2-alkyl shift to **101b** and elimination to re-form the exocyclic olefin in **99**. An elegant bioinspired synthesis of antroalbocin A (**99**) was reported by Kalesse using a photochemical rearrangement of a 5/5/6 tricyclic system to construct the bridged system [149].

**Scheme 26:**
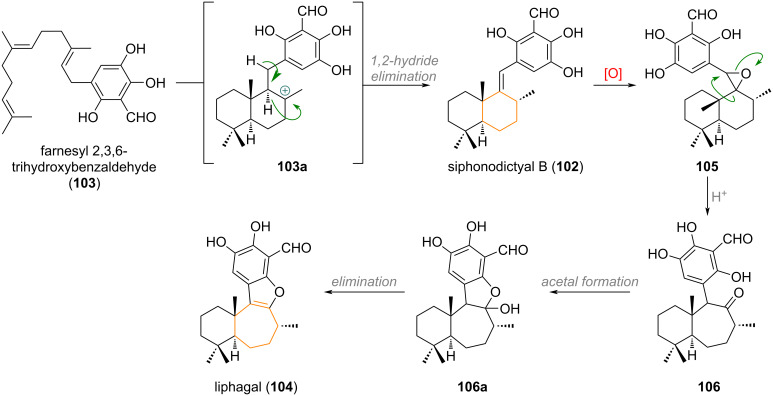
Proposed biogenetic origin for the meroterpenoid liphagal (**104**) via epoxide-mediated ring expansion.

The meroterpenoid siphonodictyal B (**102**) – presumably formed from a polyene cyclisation of chimeric precursor **103** via decalin formation **103a** and 1,2-hydride shift (see Scheme 26) – is transformed into the ring-expanded related terpenoid liphagal (**104**) [150]. Oxidation of the alkene in **102** may give rise to an oxidised species, such as **105**, which is able to undergo Meinwald rearrangement to the ring-expanded ketone **106**. From here, epimerisation of the C-8 methyl group and acetalisation to **106a** followed by elimination delivers the final natural product liphagal (**104**).

**Scheme 27:**
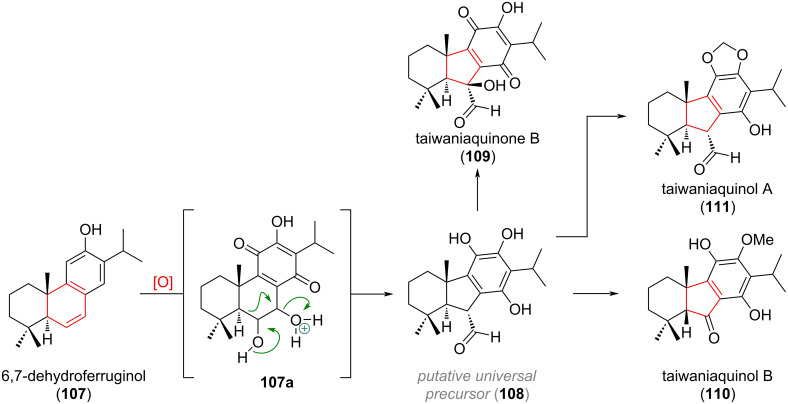
Proposed biogenetic origin for the ring-contracted members of the taiwaniaquinol family.

A ring contraction from 6→5 was suggested for the biosynthesis of taiwaniaquinol natural products and is depicted in Scheme 27 [151–156]. The precursor to this family of natural products, 6,7-dehydroferruginol (**107**), is oxidised both at the benzene core and at the pendant olefin, to give a diol **107a**, which can undergo a 1,2-alkyl shift delivering the universal precursor **108**. From here, α-oxidation and dearomatisation gives taiwaniaquinone B (**109**), oxidative deformylation taiwaniaquinol B (**110**) and methylendioxy cyclisation taiwaniaquinol A (**111**).

**Scheme 28:**
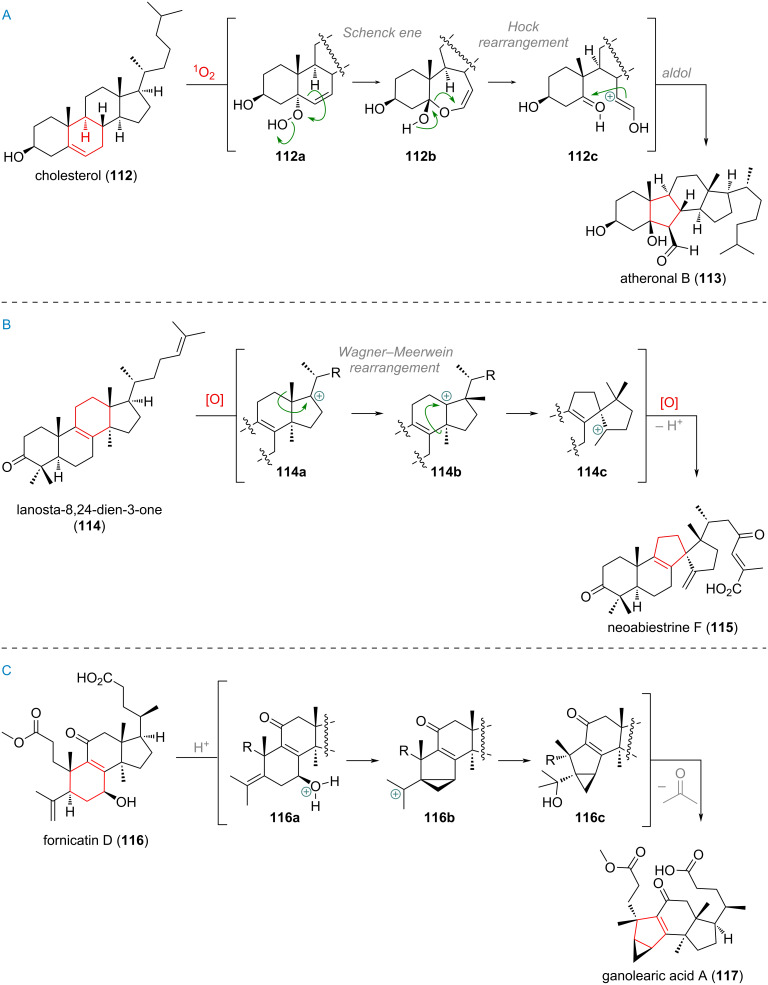
A: Schenck ene/Hock/Aldol cascade effecting B-ring contraction in atheronal B (**113**); B: Selective C-17 oxidation and double 1,2-alkyl shift build up the spirocyclic structure of neoabiestrine F (**115**); C: B-ring contraction of seco-terpenoid fornicatin D (**116**) to afford the 3/5/6/5-ring system of ganolearic acid (**117**).

The steroidal B-ring contracted compound atheronal B (**113**, see Scheme 28A) has been the topic of extensive investigation, as it was originally believed to be possibly formed inside the human body through oxidation of cholesterol (**112**) by endogenous ozone [157–158]. Pratt et al. could show that a cascade reaction, starting with a Schenck ene reaction of cholesterol to form the highly reactive hydroperoxide species **112a** was the operational pathway [159–162]. Carbon bond migration in a process called Hock cleavage leads to a cyclic hemiacetal **112b** which ring-opens and aldolises (**112c**) to give the carbaldehyde product atheronal B (**113**) with a contracted 5-membered B-ring.

The rearranged triterpenoid neoabiestrine F (**115**), corresponds to a small family of C-ring-contracted spirocyclic terpenoids which are suspected to originate from a lanostane precursor, such as **114** (see Scheme 28B) [163–165]. By way of selective oxidation at C-17 (possibly through HAT and SET-induced generation of the positive charge as described in other examples above) the C-17-centred carbocation **114a** can undergo a cascade rearrangement, consisting of an initial 1,2-methyl migration to **114b**, followed by the crucial 1,2-alkenyl migration/spirocyclisation and finally elimination at the exocyclic position to afford cation **114c**. Follow-up oxidative tailoring processes, furnishing the side-chain oxidation states of neoabiestrine F (**115**) could conceivably occur both before or after the rearrangement, though related co-isolates suggest oxidative patterns being in place already prior the change in carbon skeleton [166–172].

The unique 3/5/6/5-ring system present in ganolearic acid A (**117**, see Scheme 28C) was traced back to the related A-ring seco-terpenoid fornicatin D (**116**). The suggested reactive pathway consists of an alkene isomerisation from the propenyl-substituent at C-5, followed by protonation of the C-7 alcohol to give intermediate **116a**. From here a transannular cyclopropanation affords the cation **116b**, which upon nucleophilic attack of water provides **116c**. Expulsion of acetone completes the proposed biosynthesis of compound **117** [173–174].

**Scheme 29:**
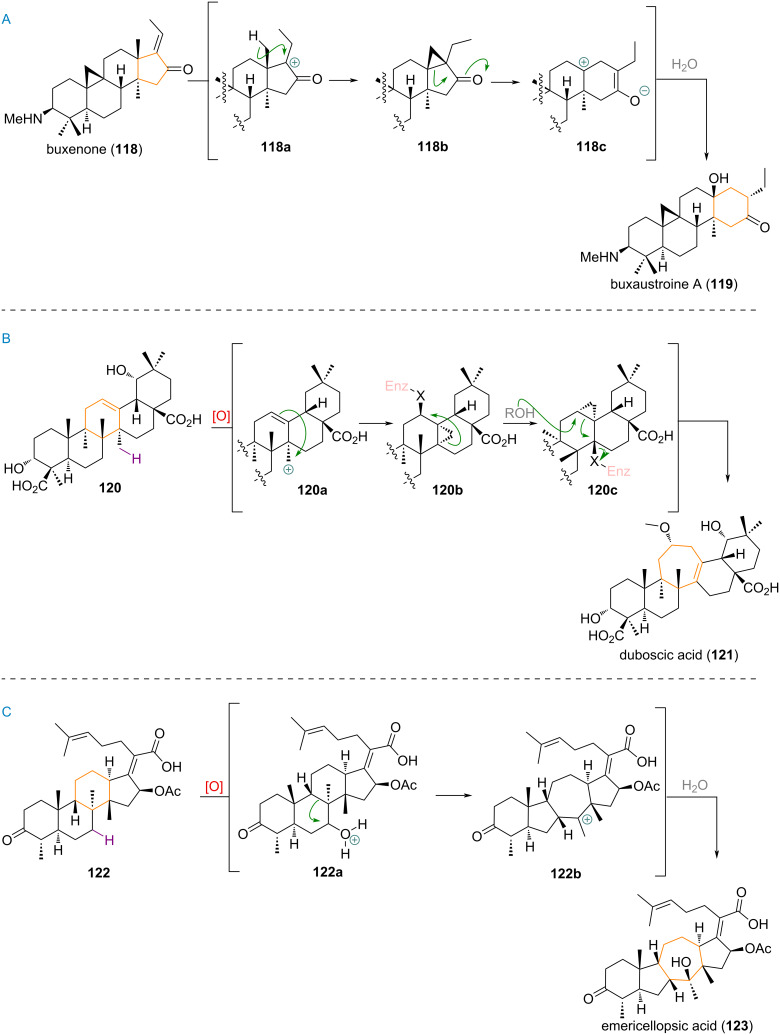
A: D-ring expansion of buxenone (**118**) via cyclopropanation towards buxaustroine A (**119**); B: Proposed ring expansion via cyclopropane intermediates in duboscic acid (**121**) biosynthesis; C: Ring-size-altering rearrangement in emericellopsic acid (**123**) biosynthesis.

Regarding steroidal products, recently an interesting example of a D-ring expansion from the common cycloartenol ring system to a novel 6/3/6/6/6 skeleton was proposed for the biosynthesis of buxaustroine A (**119**) from buxenone (**118**, see Scheme 29A) [175–176]. Formation of a carbocation **118a** at C-17 by protonation of the pendant alkene in **118** is followed by cyclopropanation, through deprotonation at the C-18 methyl group. The collapse of the cyclopropyl moiety in **118b** reveals a zwitterionic intermediate **118c**, which, upon capture of the cation by water and protonation of the enolate, delivers the rearranged natural product **119**.

An interesting C-ring-expanded triterpenoid was isolated from *Duboscia macrocarpa* in 2010 and named duboscic acid (**121**) [177–178]. It was biogenetically traced back to the oleane precursor **120** and proposed to be formed by oxidation of the C-27 methyl group (**120a**), resulting in first instance in a C-12 functionalised compound **120b** with a novel cyclopropane ring connecting C-13 and C-14 (Scheme 29B). Migration of the cyclopropyl into the C-12 position places the enzyme leaving group at C-14 (**120c**). Finally, attack of a nucleophile towards C-12 of **120c** ruptures the cyclopropane ring in a fragmentation reaction, kicking out the leaving group at C-14 in the process and expanding the 6-membered C-ring to furnish the final product **121**.

Finally, an example for a concomitant B/C-ring contraction and expansion was recently described for emericellopsic acid (**123**) and is depicted in Scheme 29C [179]. Hydroxylation at C-7 of the precursor **122** was proposed to take place, the protonation (**122a**) of which triggers a 1,2-alkyl shift of the C-8/C-9 σ-bond towards C-7 giving a tertiary carbocation **122b** which is captured by water as the nucleophile, to yield the product **123**.

**Scheme 30:**
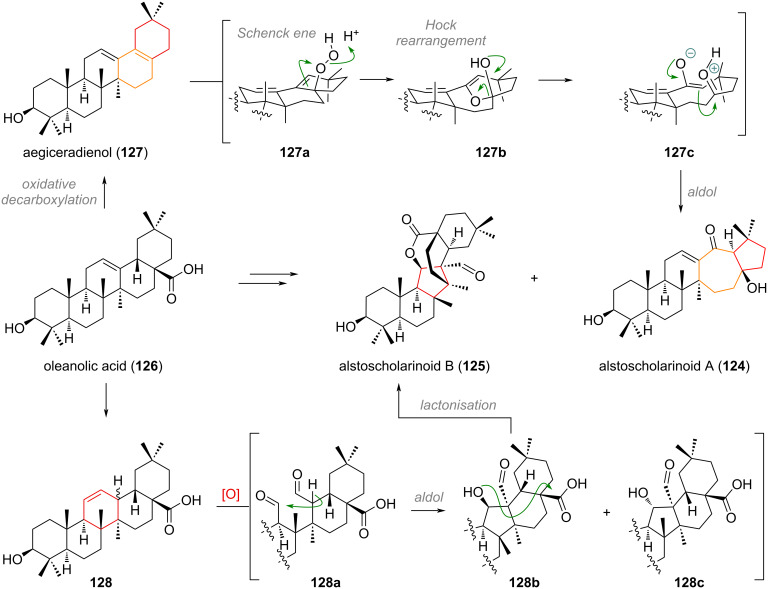
Biosynthetic origin of alstoscholarinoids A (**124**) and B (**125**) via cascade oxidative rearrangement consisting of Schenck ene/Hock/aldol reaction and intramolecular aldol/transesterification, respectively.

In 2021, two new structurally unique triterpenoids were isolated from *Alstonia scholaris*, namely alstoscholarinoids A (**124**) and B (**125**) [180–181]. Traced back to oleanolic acid (**126**), the originally proposed biosynthesis involved decarboxylation towards aegiceradienol (**127**) for compound **124** and isomerisation towards **128** for compound **125** as depicted in Scheme 30. From here an oxidative cleavage was invoked for both olefins, revealing, e.g., dialdehyde **128a**, which can engage in an aldol addition to effect ring contraction. One of the two putative isomers (**128b**) of the aldol addition is privileged to undergo intramolecular esterification to give alstoscholarinoid B (**125**). The configuration of the other isomer **128c** has been reportedly isolated already in 2002 by Kuo and Chiang [182]. For alstoscholarinoid A (**124**) an analogous oxidative cleavage and selective aldolisation (**127c**) was proposed by the isolation team, but the groups of Wu and Kratena [183–184] discovered independently that a cascade of Schenck ene/Hock/Aldol reaction (**127a** to **127b**) offers a more likely explanation for its origin, as it exclusively delivered the correct isomer **124** during bioinspired synthesis (vide infra).

**Scheme 31:**
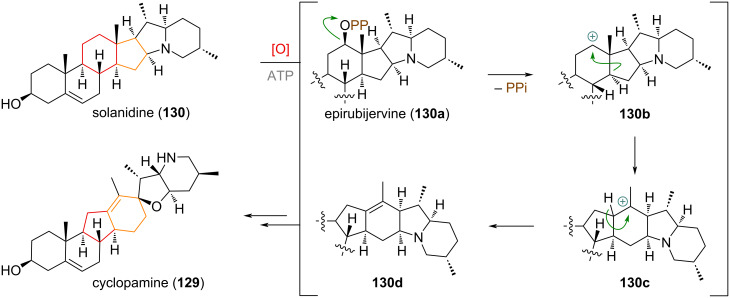
Biogenetic origin of the hedgehog signalling inhibitor cyclopamine (**129**) by tandem ring contraction/expansion of solanidine (**130**) through C-12 oxidation and Wagner–Meerwein rearrangement.

The steroidal alkaloid cyclopamine (**129**), isolated from various species of the genus *Veratrum* exhibits rearranged C and D rings (see Scheme 31). From the more classical steroidal alkaloid solanidine (**130**), which is also present in the same species, a biosynthetic reaction was proposed, starting with oxidation at the C-12 position on the C ring. The oxidised species, epirubijervine **130a**, can eliminate diphosphate to give a secondary cation **130b**, which undergoes tandem ring expansion/contraction from a 6/5 to the 5/6 system. The resulting tertiary cation **130c** is quenched by elimination of the C-13 hydrogen to form an olefin (**130d**) and furnish cyclopamine (**129**) [185–188].

**Scheme 32:**
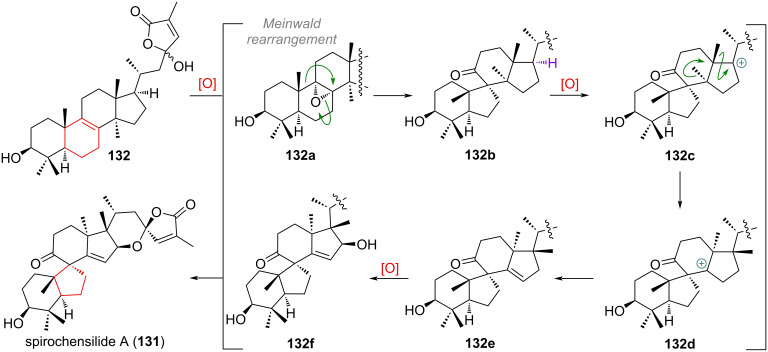
Proposed biogenetic origin of the B-ring contracted spirocyclic triterpenoid spirochensilide A (**131**).

Another interesting group of skeleton-modified triterpenoids are 6/5/6/5/6-spiro compounds like spirochensilide A (**131**, see Scheme 32) [189–191]. They are assumed to be formed by epoxidation of the 8,9-double bond present in a putative oxidised lanostane precursor **132**. Meinwald (or semi-pinacol) rearrangement of epoxide **132a** would then build up the 6/5/6-spiro system **132b** for rings A, B and C. The final product is obtained following an oxidation at C-17, multiple 1,2-methyl shifts (via cations **132c** and **132d**) and finally elimination to give **132e**, with the full skeleton assembled. After selective oxidation of the C-16 position giving **132f**, spiroacetal formation and esterification with the side-chain ketone affords spirochensilide A (**131**).

**Scheme 33:**
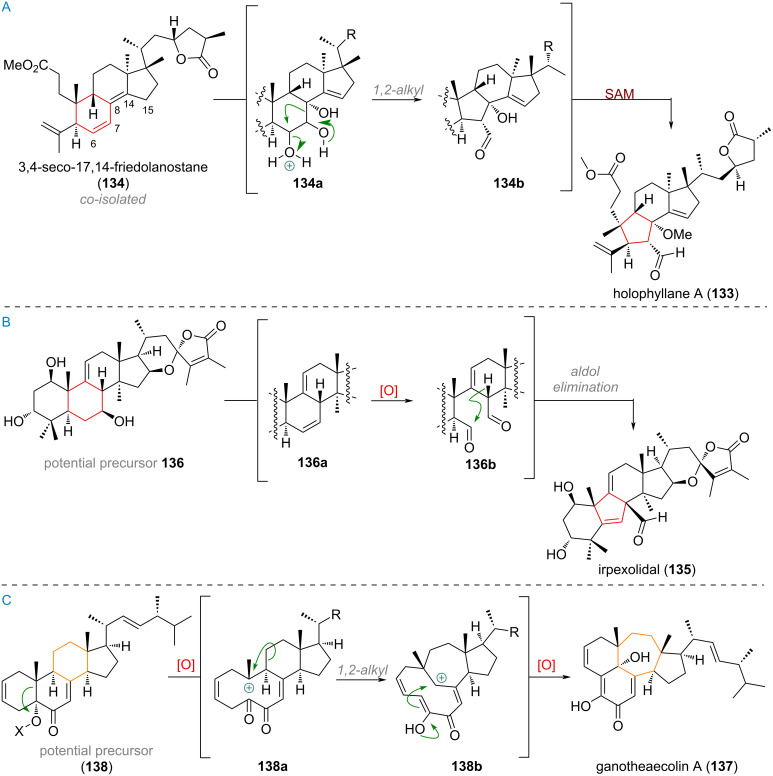
A: Proposed B-ring contraction during the biosynthesis of holophyllane A (**133**); B: B-ring contraction via aldol condensation of a dialdehyde towards irpexolidal (**135**); C: C-ring expansion via a cationic decalin system explains the biogenetic origin of ganotheaecolin A (**137**).

The A-ring-*seco* triterpenoid holophyllane A (**133**), first isolated in 2016, is a B-ring contracted triterpenoid with a markedly different proposed biosynthetic origin [192–193]. Starting from the unnamed co-isolate **134**, exhaustive oxidation is invoked to take place at the B-ring to form a triol, such as **134a** (see Scheme 33A). This can undergo semi-pinacol rearrangement, resulting in the desired 1,2-alkyl shift to form the 5-membered ring in intermediate **134b**. Alternatively, an isomerised compound with olefins in positions 7/8 and 14/15 instead of 6/7 and 8/14 could be oxidatively cleaved and undergo aldol condensation. In any case the tertiary alcohol at C-8 is methylated by way of a transmethylating enzyme and SAM (*S*-adenosylmethionine) to furnish holophyllane A (**133**).

Such an aldol reaction being responsible for B-ring contraction was reported for the formation of the B-ring-contracted triterpenoid irpexolidal (**135**) and is depicted in Scheme 33B [194]. The proposed precursor **136**, with a hydroxy group at C-7 was thought to undergo elimination to form a 6,7-olefin **136a** which can be oxidatively cleaved to, in turn, reveal dialdehyde **136b**. Aldol addition towards the C-6 aldehyde and elimination of the aldol adduct to form a 5,6-olefin delivers the product irpexolidal (**135**).

Finally, as shown in Scheme 33C, an intriguing rearrangement and C-ring expansion was proposed for the biogenesis of the highly rearranged triterpenoid ganotheaecolin A (**137**) [195–196]. First, the formation of a carbocation **138a** at C-10 is suspected, putatively formed by placing a leaving group on the C-5 tertiary hydroxy group of the precursor **138**. This intermediate is privileged to undergo a 1,2-alkyl migration expanding the C-ring, giving a different decalin cation **138b**. Keto/enol-tautomerism and nucleophilic attack of the enol at C-4/C-5 furnishes the tetracyclic system and a final oxidation at C-9 the product ganotheaecolin A (**137**).

**Scheme 34:**
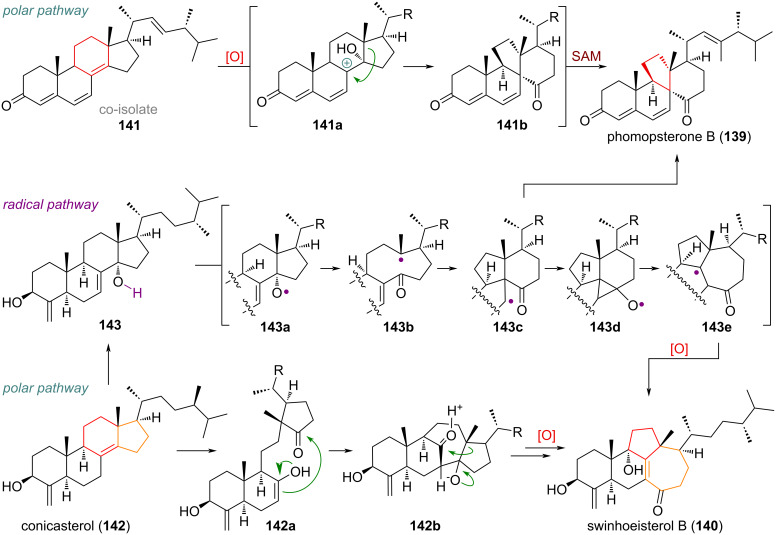
Radical and ionic/polar mechanisms for the C-ring-contracted triterpenoids phomopsterone B (**139**) and swinhoeisterol B (**140**).

The two C-ring-contracted compounds phomopsterone B (**139**) [197–199] and swinhoeisterol B (**140**) [200–201] were initially proposed to be formed by reactions with ionic mechanism, but it was shown by Heretsch [202] that the skeletal modifications observed in these natural product families can also arise through radical-initiated fragmentations (see Scheme 34). The polar pathway proposed for the dankastarone–phomopsterone family, starts with oxidation (e.g., epoxidation) of the 8,14-olefin in the co-isolate **141** and formation of an allylic cation **141a** at C-8. The 1,2-alkyl shift of the C-13/C-14 σ-bond forms the spirocyclic/bridged system **141b**, and a final methylation at the side chain by SAM finishes the biosynthesis of phomopsterone B (**139**). In turn, swinhoeisterol B (**140**) was suggested to be formed by oxidative cleavage of the 8,14-olefin present in conicasterol (**142**). The obtained diketone **142a** could undergo aldol addition to give intermediate **142b**, which upon 1,3-shift of an alkyl group would furnish the carbon skeleton of the product **140**. The alternative radical pathway suggested by Heretsch starts from a C-14 hydroxylated compound such as **143**, which is oxidised to its alkoxy radical **143a**. A β-scission event of this radical can give the C-13-centred radical **143b** which undergoes the crucial cyclisation towards C-8 to, in turn, lead to radical **143c**. Following a radical quench, this would furnish the skeleton of phomopsterones directly. Alternatively, the radical at C-7 can attack the pendant ketone to form a cyclopropyl alkoxy radical **143d**, which ring opens (**143e**) to build up the seven-membered ring of swinhoeisterol B (**140**).

**Scheme 35:**
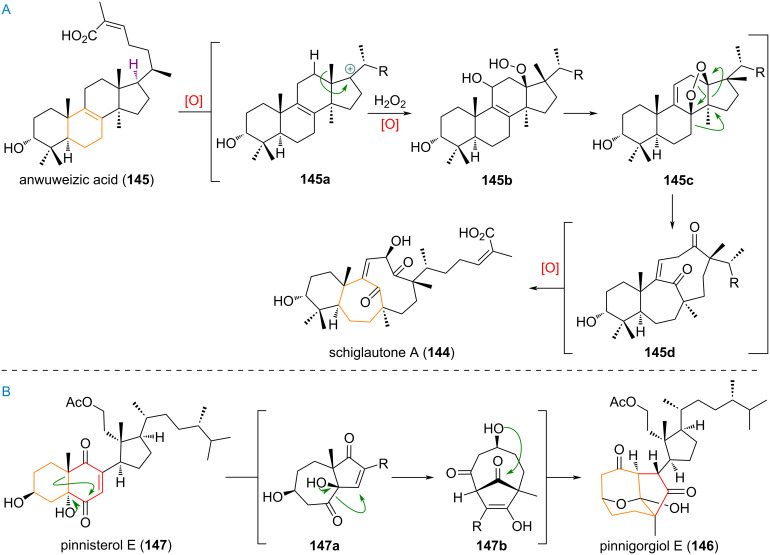
A: Plausible mechanism for the formation of schiglautone A (**144**) from anwuweizic acid (**145**); B: Proposed biosynthetic origin of pinnigorgiol E (**146**) from pinnisterol E (**147**).

The complex rearranged triterpenoid schiglautone A (**144**), was proposed to be biosynthetically formed starting from anwuweizic acid (**145**), but the proposed transformations disclosed in the original isolation report [203–204] were considered dubious and are therefore not reproduced in this review. Instead, the proposal by Werner and Kalesse is depicted [205–209]. It is conceivable that, in analogy to related compounds **114** and **131**, an initial oxidative event at C-17 (**145a**) results in the 1,2-methyl group shift (see Scheme 35A). Capture of the tertiary carbocation by a peroxy-species would deliver **145b**, which upon C-11 hydroxylation and S_N_’ substitution by the hydroperoxide could give the advanced intermediate **145c**. This substrate would now be versed to undergo a rearrangement, cleaving the C-13/C-14 σ-bond to give the diketone **145d**, which is finally oxidised at C-12, next to the ketone to furnish schiglautone A (**144**).

The highly modified *seco*-triterpenoid pinnigorgiol E (**146**), depicted in Scheme 35B, reportedly undergoes a double A-ring expansion from the 6 to an 8-membered ring from its biosynthetic precursor pinnisterol E (**147**) [210–213]. The first ring expansion comes by way of α-ketol rearrangement of the C-5 alcohol and C-6 ketone giving intermediate **147a**. From here a second 1,2-alkyl shift of the newly formed carbonyl towards the Michael acceptor in the B-ring gives the 8/5-bridged ring system **147b**. A final hemiacetal formation furnishes the tricyclic structure of pinnigorgiol E (**146**).

**Scheme 36:**
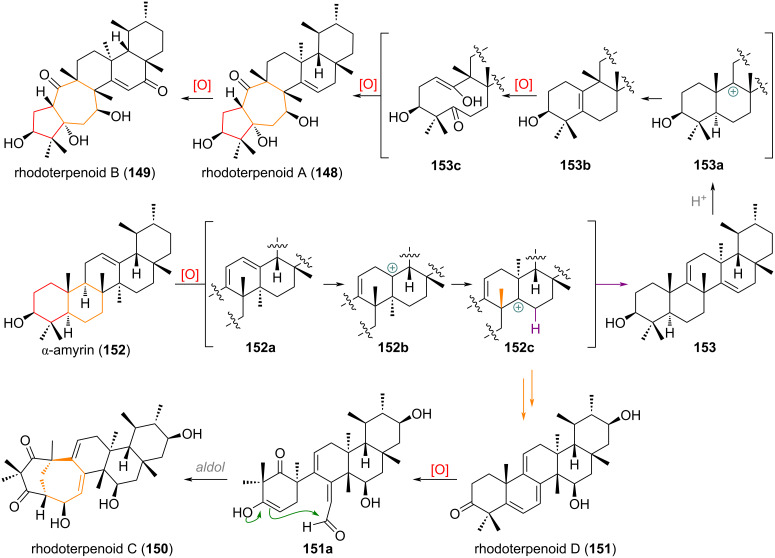
Reported biosynthetic proposal for the formation of B-ring expanded triterpenoids rhodoterpenoids A–D **148**–**151** from α-amyrin (**152**).

As a last example, the partly rearranged rhodoterpenoids A–D **148**–**151**, isolated in 2017 and traced back by the authors to the basic triterpenoid α-amyrin (**152**) [214] are discussed. From **152** an oxidation at C-11 could lead to diene **152a**, which upon protonation at C-12 forms a C-13 carbocation **152b**. Methyl group migration towards the C-14 cation opens two putative pathways (see Scheme 36). The purple pathway (oleane→taraxerane skeleton) finishes the first rearrangement with elimination from C-15 to give **153**. A second protonation event at C-11 would then give C-9 cation **153a**, poised for another 1,2-methyl shift and elimination to the 5,10-olefin **153b**. Oxidative cleavage and aldol reaction (via Hock mechanism or “normal” giving **153c**) leads to the ring-expanded/contracted rhodoterpenoid A (**148**) and another allylic oxidation at C-16 gives rhodoterpenoid B (**149**). If the orange pathway is operational, a second 1,2-methyl shift takes place immediately, giving rhodoterpenoid D (**151**) after elimination and a second dehydrogenation reaction. Oxidative cleavage of the newly formed 5,6-olefin towards **151a** and intramolecular aldol addition furnishes rhodoterpenoid C (**150**).

### Applications of ring-size-altering reactions in the total synthesis of terpenoids

Since the seminal works by Johnson [215–219], Heathcock [220–223], and Majetich [224–226] demonstrated the power of biomimetic reaction design, numerous modern total synthesis projects [11–18,227–237] have sought to leverage or evaluate proposed biosynthetic disconnections, including ring-size-altering rearrangements to great effect [238–242]. In this review, both types of examples will be included: 1) bioinspired strategies, built to test a particular biosynthetic hypothesis and 2) convenient and elegant applications of ring-size-altering reactions in natural product synthesis, regardless of biosynthetic relationship. In this section we aim to review examples in total and bioinspired synthesis which either closely resemble the proposed biosynthetic pathways, or which take inspiration from the principles that were explored in the first part of this review to effect challenging ring-size modifications. Apart from these, selected examples of interesting unrelated ring-contraction strategies (like Wolff-rearrangement) have been included.

**Scheme 37:**
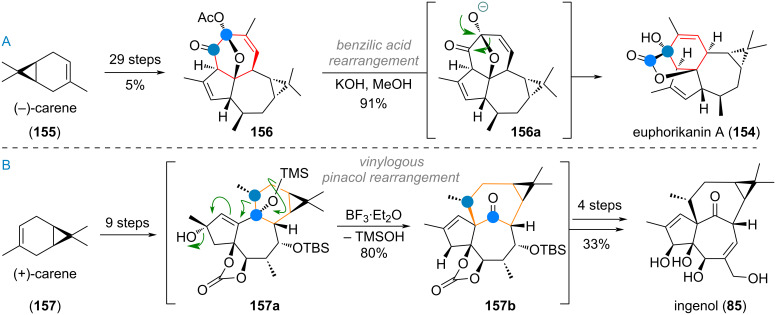
A: Final reaction step in the synthesis of euphorikanin A (**154**), benzilic acid-type ring contraction reported by Jia and co-workers; B: bioinspired vinylogous pinacol rearrangement in Baran’s synthesis of ingenol (**85**).

The Jia group reported their bioinspired synthesis of euphorikanin A (**154**) starting from (−)-carene (**155**), already containing the dimethyl cyclopropane motif [243–244]. Intermediate **156** was reached after 29 linear steps and suggested to be the crucial biosynthetic precursor (protected as its acetate, see Scheme 37A). Treatment with strong alkali cleaved the ester in **156**, revealing oxyanion **156a**, which underwent a 1,2-alkyl shift in a benzilic acid rearrangement to give the bridged lactone ring of euphorikanin A (**154**).

Likewise, Baran et al. reported a synthesis of the ingenane diterpenoid ingenol (**85**, see Scheme 37B) starting from (+)-carene (**157**) which was elaborated to the complex tricyclic intermediate **157a** in just 9 linear steps [245]. This intermediate closely resembles the suggested biosynthetic intermediates for the tigliane→ingenane transformation in nature. Pleasingly, when **157a** was treated with strong Lewis acids, a vinylogous pinacol rearrangement proceeded smoothly to give the ring-expanded bridged system intermediate **157b**. Further oxidation and carbonate deprotection furnished ingenol (**85**) in only 4 more steps.

**Scheme 38:**
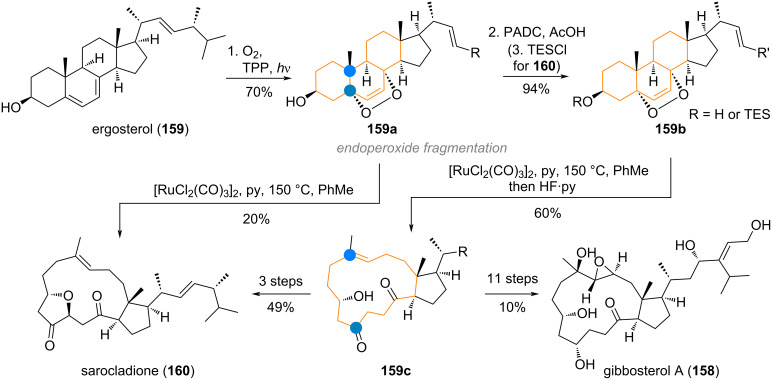
Tricyclic ring expansion in the Gui synthesis of gibbosterol A (**158**) and sarocladione (**160**) via Ru-catalysed endoperoxide fragmentation.

The structurally intriguing highly oxidised and ring-ruptured steroidal natural product gibbosterol A (**158**) was targeted by the Gui group [246] by means of a bioinspired strategy [247]. The crucial rupture of both A/B and B/C ring junctures in a single step was realised by formation of the bridged endoperoxide **159a** from ergosterol (**159**, see Scheme 38), followed by hydrogenation/protection to give **159b**. Both intermediates **159a** and **159b**, could then be fragmented under ruthenium catalysis to give either sarocladione (**160**) or intermediate **159c**, which could be carried over to gibbosterol A (**158**) in another 11 steps, mainly consisting of adjusting oxidation states and attaching the correct side-chain residues.

**Scheme 39:**
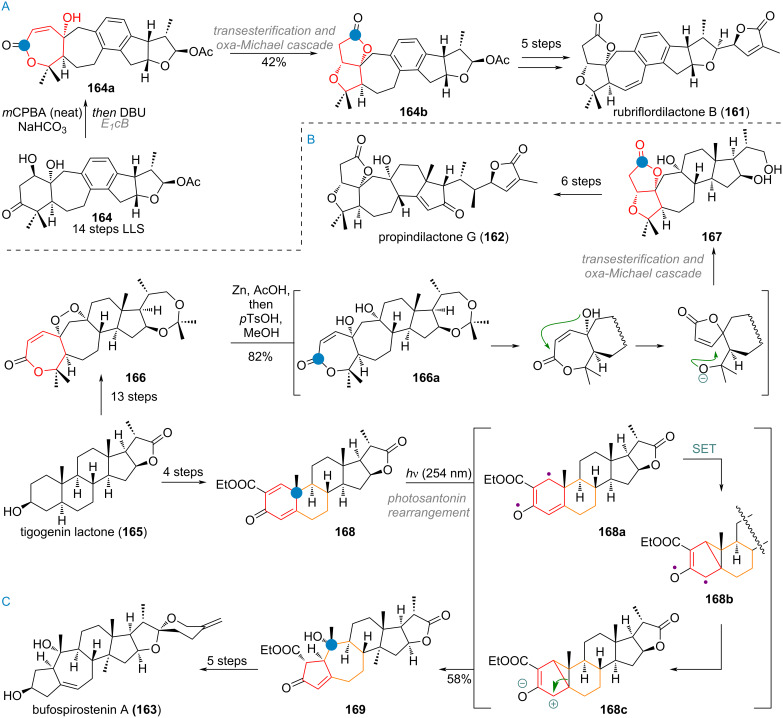
A: A-ring expansion during the Gui synthesis of rubriflordilactone B (**161**); B: Mechanism for the bioinspired A-ring contraction of a 7-membered A-ring lactone to 5-membered lactone of propindilactone A (**162**); C: Tandem ring expansion/contraction of a santonin analogue in Gui’s synthesis of bufospirostenin A (**163**).

The Gui group also reported multiple syntheses of triterpenoids with rearranged A/B-ring connectivity, such as rubriflordilactone B (**161**) [248], propindilactone G (**162**) [249] and bufospirostenin A (**163**, see Scheme 39) [250], compounds which have gathered a lot of interest and have been chosen as targets for total synthesis from various groups [251–261]. For the product **161**, the advanced intermediate **164** obtained after 14 steps undergoes a Baeyer–Villiger oxidation towards the 7-memberd lactone **164a** with concomitant E1cB elimination of the β-hydroxy group (see Scheme 39A). A transesterification/oxa-Michael addition mechanism is then responsible for the rearrangement of the A-ring towards the 5-membered lactone **164b**. Finally, oxidation state adjustment at the benzylic position and side-chain elaboration furnished rubriflordilactone B (**161**).

The other two semi-syntheses of this family of triterpenoids by the Gui group started from tigogenin lactone (**165**), an abundant and commercially available steroid starting material. It was transformed into endoperoxide **166** in 13 steps, before the crucial bioinspired A-ring cascade was performed (see Scheme 39B). Reduction of the peroxide with elemental zinc under acidic conditions triggered transesterification in **166a** by the tertiary hydroxy group at C-10 followed by intramolecular oxa-Michael ring closure towards **167**. This advanced intermediate was carried over to the final product **162** in 6 additional steps. Finally, Gui and co-workers also applied the photosantonin rearrangement (vide supra, Scheme 12) to the synthesis of A/B-ring contracted compounds, such as **163**. Thus, the A-ring of tigogenin lactone (**165**) was exhaustively oxidised to give dienone ester **168**, which cleanly underwent the desired rearrangement via **168a**–**c** to give the 5/7-ring system of **169**. The desired natural product **163** was reached in just 5 additional steps, highlighting the efficiency of bioinspired semisynthetic approaches (see Scheme 39C).

**Scheme 40:**
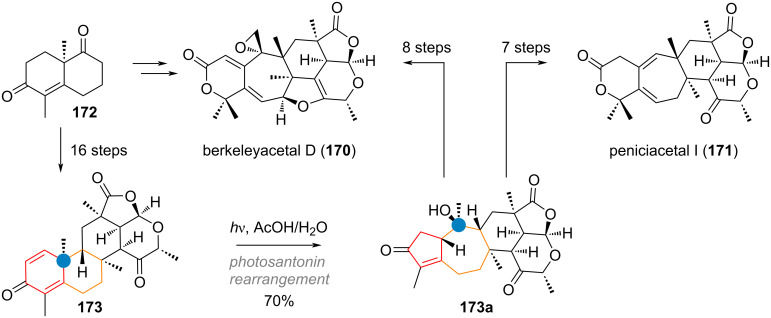
Photosantonin rearrangement effects A/B ring contraction/expansion in Li’s synthesis of the complex meroterpenoids berkeleyacetal D (**170**) and peniciacetal I (**171**).

Recently, Li and co-workers reported elegant syntheses of the highly complex DMOA-based meroterpenoids berkeleyacetal D (**170**) and peniciacetal I (**171**) [262]. After reaching the advanced intermediate **173** from the decalin **172** in 16 steps the photosantonin rearrangement was exploited to expand the B-ring from 6→7 and to contract the A-ring to eventually transform it into the 6-membered lactone present in the final products (see Scheme 40). After another 7 and 8 steps, respectively, from enone **173a** the target natural products **170** and **171** with the 6/7/6/5/6 pentacyclic frameworks were obtained.

**Scheme 41:**
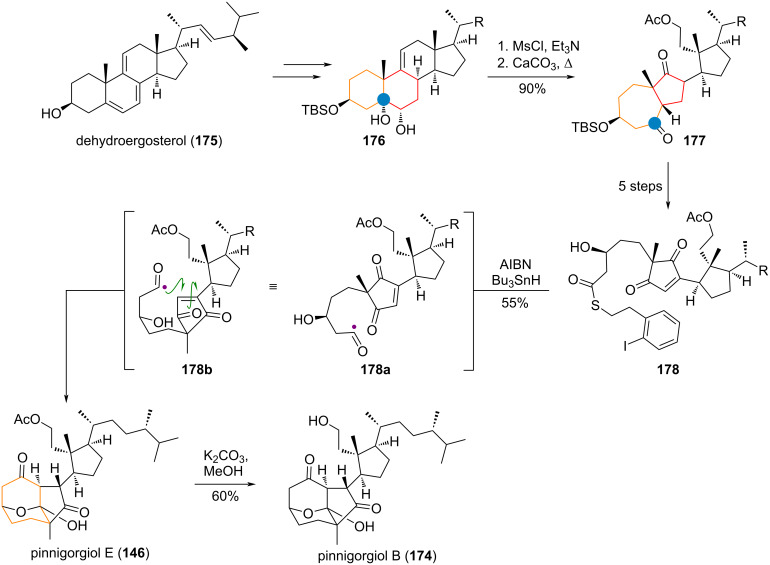
Tandem A/B ring expansion/contraction of an ergosterol derivative via pinacol rearrangement in the synthesis of pinnigorgiols B (**174**) and E (**146**) reported by Gui et al.

During the synthesis of pinnigorgiols B (**174**) and E (**146**) from dehydroergosterol (**175**, see Scheme 41) by the Gui group, two A-ring expansions were performed [263–264]. The first, a classic pinacol rearrangement of a secondary/tertiary diol **176**, was performed by mesylation followed by treatment with base at high temperature, giving the desired 7/5-ring system in **177**. The bond between C-5 and C-6 was cleaved and carried over to thioester **178** in 5 steps. The acyl radical generated from this species undergoes a Michael addition towards the Michael acceptor, resulting in a de facto multistep ring expansion to the 8-membered carbocycle. Thus, pinnigorgiol E (**146**) was obtained and deacetylated to give the sister natural product pinnigorgiol B (**174**).

**Scheme 42:**
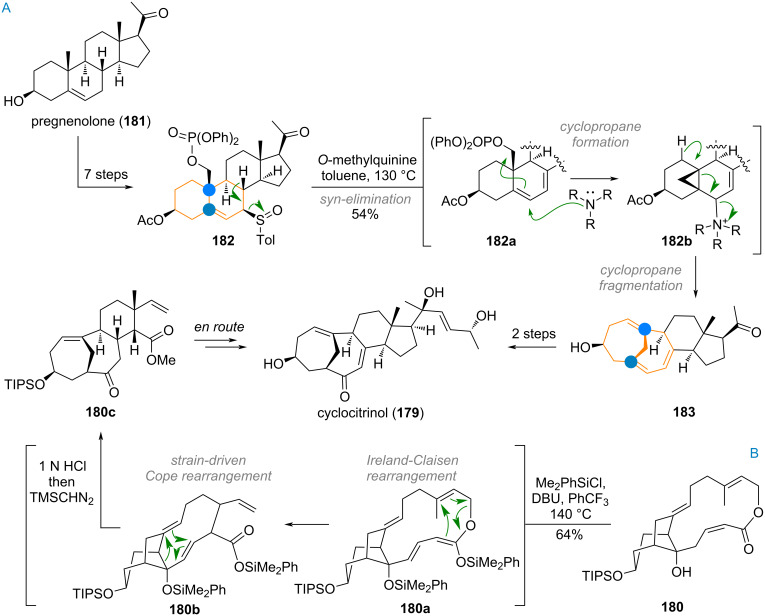
Synthetic studies towards cyclocitrinol (**179**) by A) the semisynthetic approach by Gui et al. using a cyclopropanation/fragmentation strategy and B) by using cascade sigmatropic rearrangements reported by Leighton.

The bridged 7/7/6/5-skeleton of the triterpenoid cyclocitrinol (**179**) has attracted the attention of multiple research groups (see Scheme 42B) [265–268]. The Leighton group [268] tackled the problem by synthesizing macrocyclic lactone **180**, designed to undergo a cascade reaction consisting of an Ireland–Claisen rearrangement (intermediate **180a**), then Cope rearrangement (**180b**) to furnish the tricyclic ABC-ring system of cyclocitrinol (**180c**).

In line with their other synthetic approaches, the Gui group wanted to tackle cyclocitrinol by double ring expansion, incorporating the C-10 methyl group as the bridging carbon [269–270]. Starting from pregnenolone (**181**) and following functionalisation of the methyl group and C-7 position in 7 steps precursor **182** was obtained (see Scheme 42A). Forced *syn*-elimination of the C-7 sulfoxide afforded double bonds in the “ergosterol” configuration. In the same pot, the tertiary amine base (quinine derivative) enables cyclopropanation of **182a** to give **182b**. Fragmentation of the cyclopropane under expulsion of the ammonium leaving group gave an advanced intermediate **183** which was carried over to cyclocitrinol (**179**) in two more steps.

**Scheme 43:**
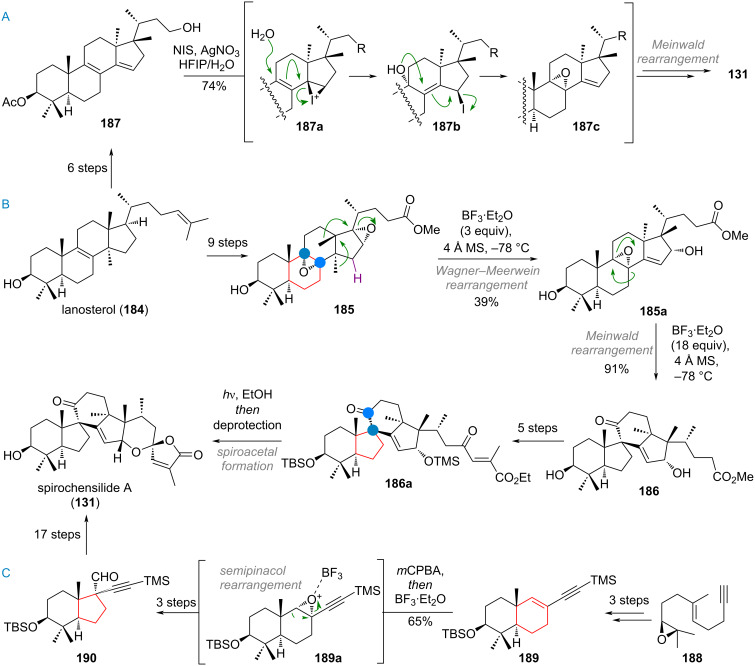
A: Bioinspired synthesis of spirochensilide A (**131**) by the Heretsch group via selective 8,9-epoxidation and rearrangement; B: Bioinspired approach by the Deng group, using a cascade double epoxide rearrangement; C: Yang’s total synthesis of **131** including a ring contraction of the B-ring via semipinacol rearrangement.

The synthesis of the complex B-ring-contracted spirocyclic triterpenoid spirochensilide A (**131**) has to date been completed by three research groups, two of which used closely related, bioinspired approaches. Starting from lanosterol (**184**, see Scheme 43B) the Deng group opted to synthesise bisepoxide **185** in 9 steps [271]. Treatment of this compound with boron trifluoride triggered opening of the 16,17-epoxide and concomitant double 1,2-methyl shift and elimination to **185a**. Treating this intermittent 8,9-epoxide with excess of the same Lewis acid triggered a second rearrangement (epoxide opening and 1,2-alkyl shift of C-7 towards C-9). The resulting product **186** was elaborated to (*E*)-configured ester **186a** in 5 additional steps; the olefin was then photoisomerised to the (*Z*)-configuration and spiroacetalisation was carried out to deliver the desired product **131**.

The Heretsch group’s approach was closely related, opting for carrying out the D-ring rearrangement first (see Scheme 43A), reaching diene **187** in just 6 steps from lanosterol (**184**) [272]. An intriguing selective epoxide formation, mechanistically explained by iodonium formation and hydrolysis, as depicted in the sequence of intermediates **187a** via **187b**, gave epoxide **187c**. The synthesis toward the desired product **131** now just required the analogous Meinwald rearrangement and attachment of the side-chain residues. Instead of trying to emulate the biosynthetic pathway, the Yang group opted for a convergent approach, preparing the AB-ring system of the targeted product from decalin **189** (see Scheme 43C, accessed in 3 steps from epoxide **188**). The 6→5 contraction of what would eventually be the B-ring was once again realised by Meinwald rearrangement of the corresponding epoxide **189a** to give the aldehyde **190** in 3 steps. From here, the desired target spirochensilide A (**131**) was reached in 17 additional steps [273–275].

**Scheme 44:**
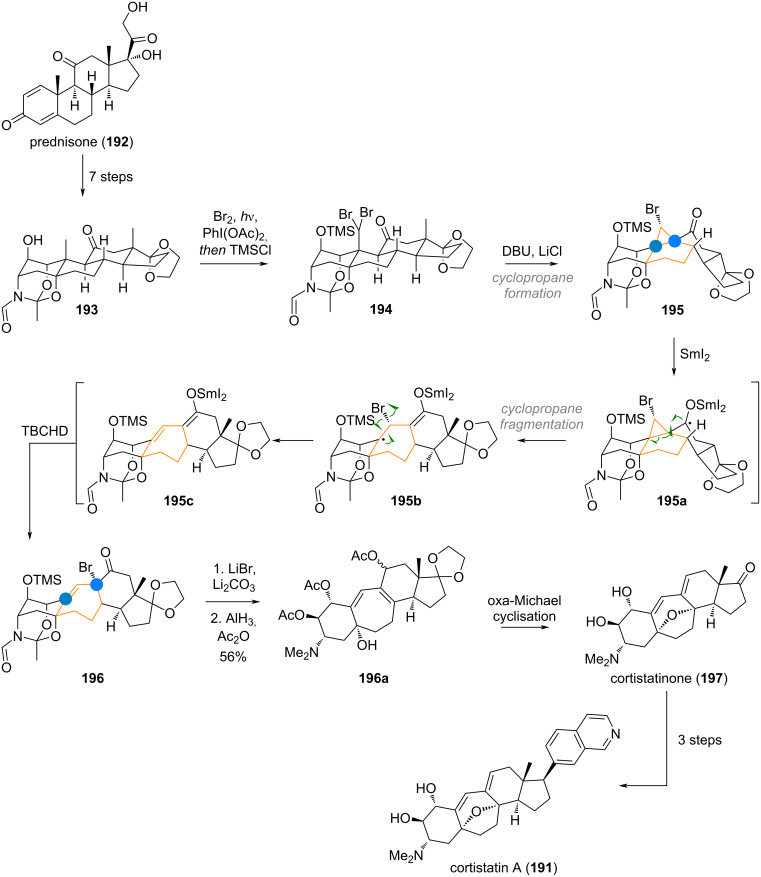
Baran’s synthesis of cortistatin A (**191**), expanding the B-ring through a cyclopropane fragmentation.

In their campaign towards the 6/7/6/5 ring system of cortistatin A (**191**), the Baran group opted for a skeletal editing approach, starting with a steroidal precursor [276–277]. Thus, prednisone (**192**) was elaborated to alcohol **193**, which was exploited for a directed radical bromination of the C-10 methyl group to give dibromide **194** (see Scheme 44). Cyclopropanation by way of intramolecular alkylation of the ketone at C-11 gave the bromocyclopropane **195**. Treatment of this intermediate with samarium(II) iodide triggered rupture of the cyclopropane (in **195a**), giving the C-10 centred radical **195b**. Expulsion of a bromine radical via **195c** followed by bromination of the samarium(III) enolate in **195c**, resulted in the ring-expanded product **196**. From here, cortistatinone (**197**) was obtained after elimination and reduction with alane (**196a**) and oxa-Michael cyclisation. The desired target compound cortistatin A (**191**) was reached in three additional steps.

**Scheme 45:**
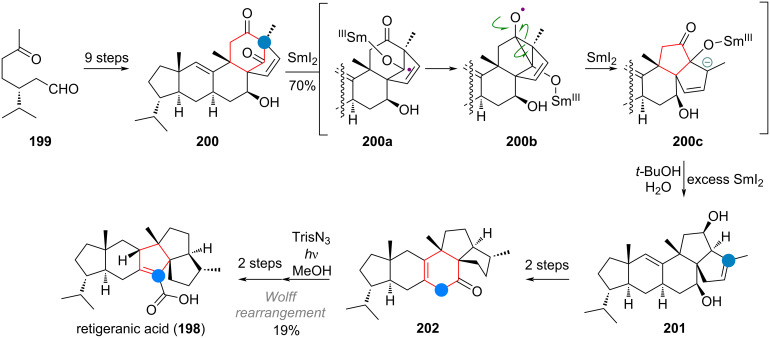
Ding’s total synthesis of retigeranic acid (**198**) showcasing sequential 6→5 ring contractions.

The total synthesis of retigeranic acid (**198**) by Ding and co-workers also exploited ketyl radical chemistry to affect ring size. In this case aldehyde **199** was carried to advanced intermediate **200** in just 9 steps [278]. Treatment with SmI_2_ leads to ketyl radical **200a**, which attacks the neighbouring ketone to form a cyclopropyloxyl radical intermediate **200b** (see Scheme 45). Opening of the cyclopropane reveals the 6/5/5 ring system. A second equivalent of SmI_2_ could conceivably form a carbanion (**200c**), which after protonation and exhaustive reduction by excess samarium reagent delivers the diol **201** as the product. This diol is carried over to ketone **202** and a diazo group is then introduced in the α-position. Irradiation then resulted in the second 6→5 ring contraction of this synthesis via Wolff rearrangement. From here retigeranic acid (**198**) was prepared in 2 further steps.

**Scheme 46:**
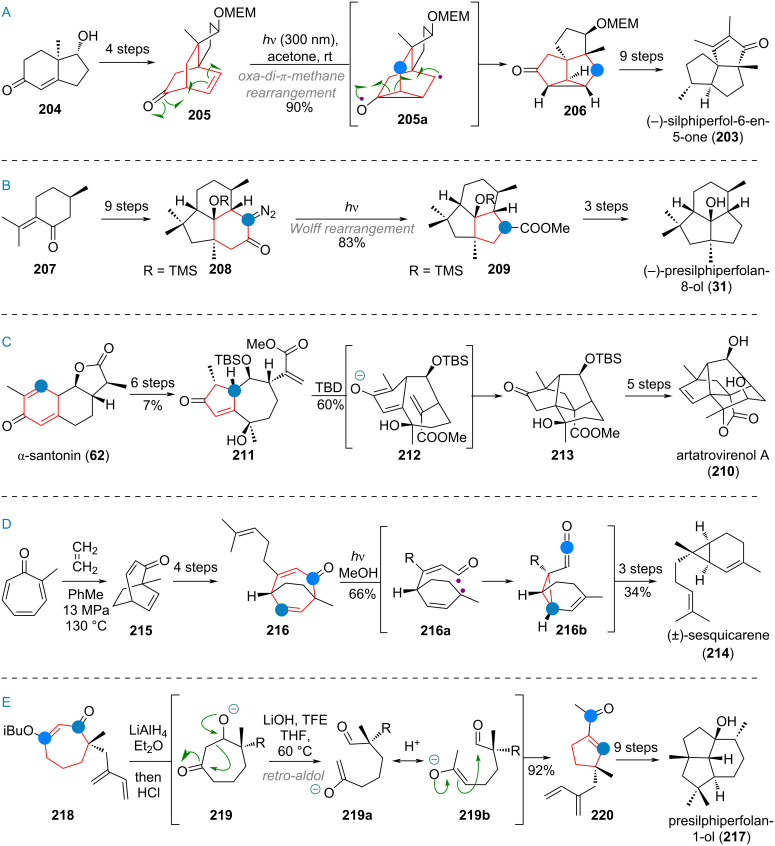
A: Oxa-di-π-methane (ODPM) rearrangement of a bicyclic ketone en route to silphiperfolenone (**203**); B: Wolff rearrangement for ring contraction en route to presilphiperfolanol (**31**); C: Ring contraction of α-santonin (**62**) in the total synthesis of artatrovirenol A (**210**); D: Norrish type 1 photocatalytic ring contraction in the total synthesis of sesquicarene (**214**); E: Retro-aldol/aldol cascade leading to ring contraction during the synthesis of presilphiperfolanol (**217**).

The synthesis of the triquinane terpenoid silphiperfol-6-en-5-one (**203**, see Scheme 46A) featured an interesting ring contraction via the rare oxa-di-π-methane rearrangement [279]. Chiral enone **204** was elaborated to give bicycle **205** in 4 steps. From here, irradiation at 300 nm triggered cyclopropane and diradical formation (**205a**). Recombination of these radicals, re-establishing the C–O double bond, gave product **206**, with the triquinane skeleton fully realised. This intermediate was carried to the product **203** in 9 steps. The related 6/5/5 tricyclic terpenoid presilphiperfolan-8-ol (**31**) was accessed from (*R*)-pulegone (**207**) by synthesizing the corresponding 6/6/5 ring system initially (Scheme 46B). The α-diazoketone **208** was then irradiated to initiate Wolff rearrangement, leading to ring-contracted ester **209**. In three additional steps the authors were able to access the target natural product (**31**) [280].

During the synthesis of the complex caged terpenoid artatrovirenol A (**210**) by the She group, the ring contraction by photosantonin rearrangement was exploited to transform α-santonin (**62**) to the IMDA precursor **211** (see Scheme 46C). Enolisation by a strong base revealed diene **212**, which underwent cycloaddition to the cage-like structure **213**. The desired target artatrovirenol A (**210**) was accessed in 5 more steps [281]. An interesting, unusual ring contraction was also reported during the synthesis of racemic sesquicarene (**214**). Methylated tropolone was subjected to [5 + 2] cycloaddition to give **215** which was then elaborated to the 7-membered bicyclic compound **216** (Scheme 46D). Irradiation leads to diradical formation by Norrish type I pathway to give intermediate **216a**. Allylic isomerisation of the acyl radical results in cyclopropane formation to give the ketene compound **216b**, which was quenched with methanol. From here, the product **214** was reached in three additional steps [282]. Finally, Scheme 46E shows the synthesis of presilphiperfolanol (**217**) which features a multistep 7→5 ring contraction. Cycloheptane **218** was reduced, then the masked ketone hydrolysed. The resulting β-alkoxy ketone **219** undergoes a retro-aldol reaction to give enolate **219a**, which can scramble to give enolate **219b**. Kinetically fast 5-ring cyclisation is now favoured and leads to the cyclopentane present in **220**. From there, 9 additional steps were necessary to reach the desired target [283–284].

**Scheme 47:**
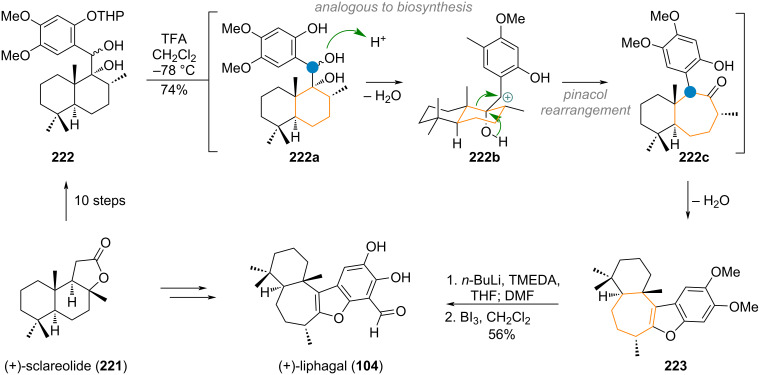
Biomimetic synthesis of liphagal (**104**) from sclareolide (**221**) by George and co-workers.

George’s synthesis of liphagal (**104**), closely followed the proposed biosynthesis, laid out above (vide supra, Scheme 26). (+)-Sclareolide (**221**), was chosen as commercially available starting material, and elaborated to diol **222** in 10 steps [285–288]. Treatment of the diol with a strong acid at low temperatures resulted in protonation of the secondary alcohol (intermediate **222a**) and formation of benzylic cation **222b**. Then, a Pinacol rearrangement, expanding the six-membered ring, takes place to deliver ketone **222c**. Finally, condensation of the concomitantly deprotected phenol with the ketone furnishes the tetracyclic core **223** of liphagal, which was obtained after two further steps (see Scheme 47).

**Scheme 48:**
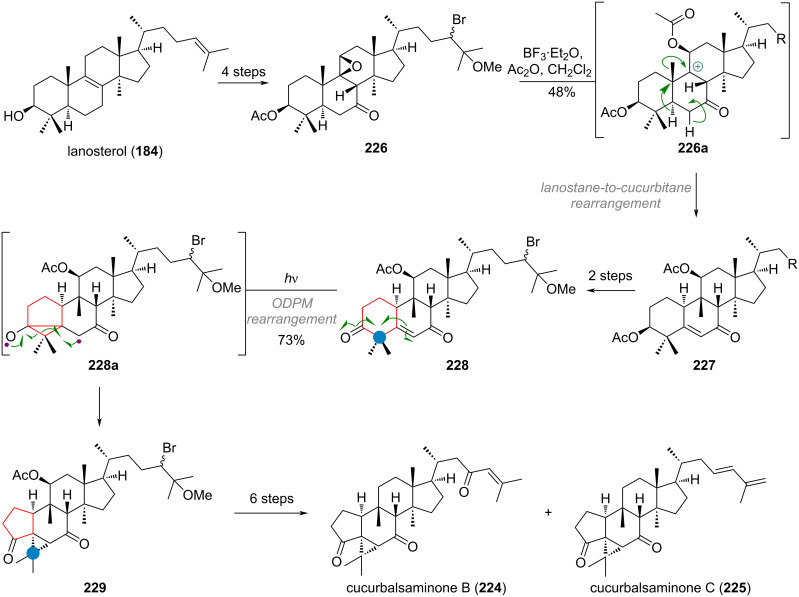
Wu’s bioinspired synthesis of cucurbalsaminones B (**224**) and C (**225**) by photocatalytic oxa-di-π-methane (ODPM) rearrangement.

The interesting A-ring-contracted triterpenoids named cucurbalsaminones **224** and **225** were suggested to be formed from the ODPM-rearrangement of an α,β-unsaturated ketone. Starting from lanosterol (**184**), Wu’s group commenced their synthesis with setting up the first skeletal rearrangement, to transform the lanostane to the cucurbitane skeleton, by a 1,2-shift of the C-10 methyl group [289–290]. Thus, epoxide **226** was synthesised in 4 steps, and treated with boron trifluoride, triggering the desired Wagner–Meerwein rearrangement and elimination along C-5/C-6, in conjugation with the ketone in **226a**. Deprotection and oxidation of compound **227** afforded diketone **228**, which, under UV irradiation, formed cyclopropane diradical **228a**. Recombination of **228a**, as depicted in Scheme 48, then directly afforded the 5/6/3-ring system from the previous 6/6 core structure.

The ring-contracted intermediate **229** was then further transformed in 6 steps to give the two compounds **224** and **225**, both members of the natural products in this family (all differing on the side-chain only) after deoxygenation of the C-11 acetoxy group. A very closely related synthesis, opting for the same strategy of lanostane→cucurbitane rearrangement and ODPM-rearrangement was recently disclosed by the Dethe group for the third natural product, cucurbalsaminone A (not depicted in Scheme 48) [291].

**Scheme 49:**
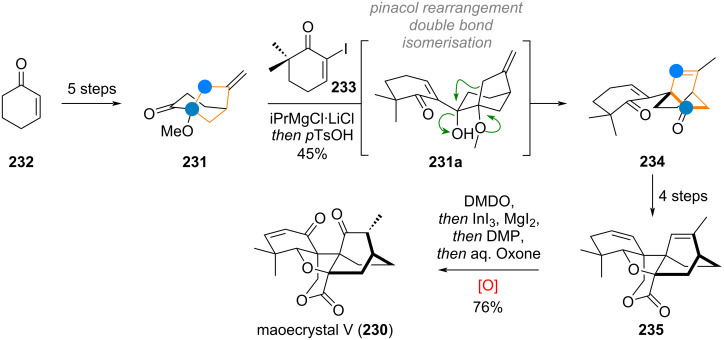
Baran’s total synthesis of maoecrystal V (**230**) featuring a pinacol rearrangement for ring expansion in a bridged bicyclic system.

The Baran group reported an interesting ring-expansion approach to the [2.2.2]bicyclooctane system present in maoecrystal V (**230**) [292–293]. When bridged cyclohexyl ketone **231** (prepared from cyclohexanone **232** in 5 steps, see Scheme 49) was attacked by the vinyl iodide **233** (after metalation) the resulting intermediate **231a** cleanly underwent acid-catalysed pinacol rearrangement, removing the original ketone oxygen and expanding the 5-membered ring into the [2.2.2]bicyclooctane system present in **234**. Additionally, the double bond migrated into the endocyclic position during this transformation. After 4 additional steps, the complete skeleton **235** of the complex terpenoid had been assembled. This material was then carried to the natural product **230** through a sequence consisting of epoxidation, epoxide opening, rearrangement (1,2-hydride shift), and elimination of iodide.

**Scheme 50:**
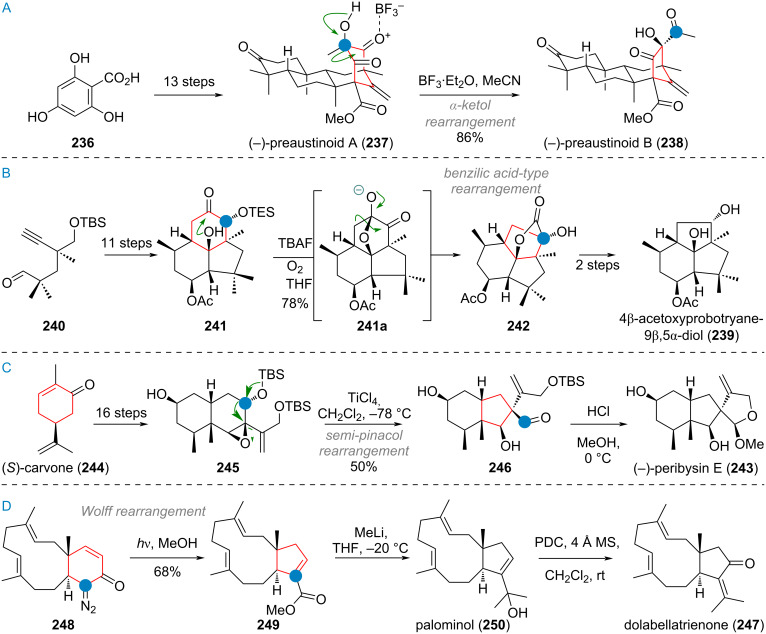
A: Ketol rearrangement leading to ring contraction in the total synthesis of preaustinoid B; B: Benzilic acid rearrangement enabling 6→5 ring contraction in the synthesis of probotryane natural product **239**; C: Semi-pinacol rearrangement to effect ring contraction in the synthesis of peribysin E (**243**); D: 6-membered ring contraction via Wolff rearrangement for the synthesis of dolabellatrienone (**247**).

Houhua Li and co-workers reported the synthesis of several members of the preaustinoid family of meroterpenoids (see Scheme 50A) [294]. Starting from 2,4,6-trihydroxybenzoic acid (**236**), preaustinoid A (**237**) was built up in just 13 linear steps. The suspected biogenetic relationship between the bridged 6/6/6/6-ring system of this product and its sister natural product preaustinoid B (**238**), was pinpointed to arise from an α-ketol rearrangement. This bioinspired step could be reproduced with great effect in the laboratory by using boron trifluoride as Lewis acid, effecting the ring contraction towards the 5-membered ring of preaustinoid B (**238**).

The group of C.-C. Li reported a similar 6-membered-ring contraction during their synthesis of the highly strained sesquiterpenoid 4β-acetoxyprobotryane-9β,15α-diol (**239**) [295]. From the starting aldehyde **240**, a 6/6/5 tricyclic system **241** was constructed in just 11 linear steps (see Scheme 50B). Fluoride-mediated TES-deprotection and autoxidation by ambient oxygen furnished the masked α-diketone **241a**, which underwent benzilic acid-type rearrangement towards lactone **242**, with a contracted carbocycle. After the final reduction and decarboxylation of this lactone moiety, the desired compound **239** was obtained.

Danishefsky’s group demonstrated a ring contraction during their synthesis of peribysin E (**243**) [296]. Starting from (*S*)-carvone (**244**) a bicyclic epoxide **245** was synthesised in 16 steps (see Scheme 50C). Treating the silyl ether **245** with titanium tetrachloride resulted in opening of the epoxide and 1,2-alkyl shift, accompanied by cleavage of the TBS group. The resulting 6/5-ring system of aldehyde **246** was transformed into the 6/5/5-spiro-acetal of peribysin E (**243**) by treatment with HCl.

Corey and Snyder utilised the Wolff rearrangement for another 6→5 ring contraction, en route to dolabellatrienone (**247**) [297]. The diazo precursor **248** was irradiated in methanol to afford methyl ester **249**, which was alkylated to give palominol (**250**). Following the transposition of the oxygen via Babler oxidation, the synthesis of dolabellatrienone (**247**) was completed (see Scheme 50D).

**Scheme 51:**
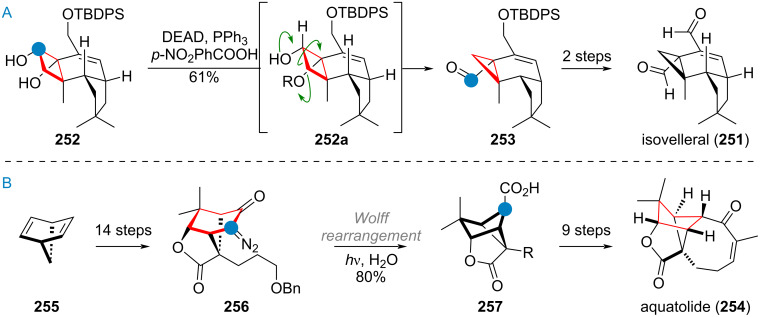
A: Scheidt’s synthesis of isovelleral (**251**) by pinacol rearrangement triggered by Mitsunobu conditions; B: Wolff rearrangement leading to ring contraction of the bridged precursor en route to aquatolide (**254**).

The Scheidt group, during the synthesis of isovelleral (**251**), accessed a terpenoid with a rare 5/6/3-ring system and constructed the crucial cyclopropane ring by a 1,2-shift and ring contraction of a 4-membered ring by means of a Mitsunobu reaction [298]. The diol **252** was reacted with DEAD and PPh_3_, triggering a pinacol rearrangement (via **252a**) which gave the tricyclic aldehyde **253**. The synthesis of isovelleral (**251**) then concluded with TBDPS deprotection and oxidation to the aldehyde (see Scheme 51A).

During the synthesis of aquatolide (**254**), Gu and co-workers used 2,5-norbornadiene (**255**) as the starting material which was elaborated to the polycyclic α-diazoketone **256** in 14 steps [299]. Carrying out the Wolff-rearrangement under aqueous conditions gave rise to the 5-memeberd carboxylic acid **257**, which already contained most of the connectivity of aquatolide (**254**), with an additional 9 steps being required to close the 8-membered ring (see Scheme 51B).

**Scheme 52:**
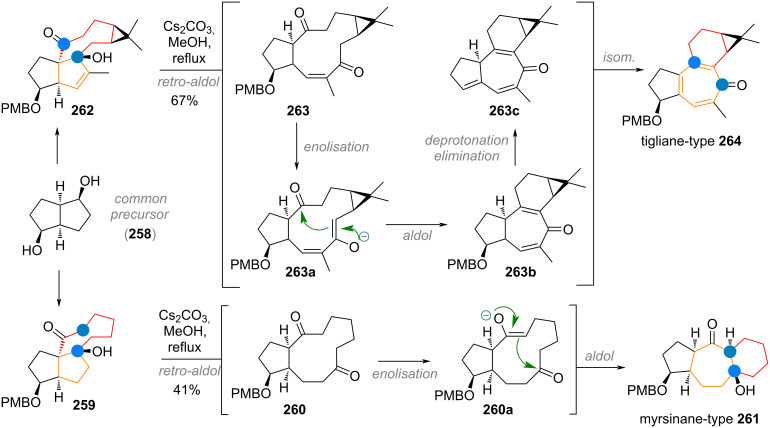
Biomimetic transformations of simplified test substrates related to Euphorbia diterpenoids.

The She group recently explored the interconnectivity of the different carbon skeleta of *Euphorbia* diterpenoids (see Scheme 52) using a simplified model [300]. Starting from the bicyclic diol **258**, two different 5/5/8-membered ring systems (related to pepluanol natural products) were synthesised and treated under basic conditions in methanol. The compound **259**, without the dimethylcyclopropane moiety underwent retro-aldol reaction to the 5/11-bicycle **260**, which can undergo opposite enolisation at the northern ketone (**260a**) and aldolise to afford compound **261**, exhibiting the myrsinane skeleton. The substrate **262**, with the pendant dimethylcyclopropane and the endocyclic olefin underwent a retro-aldol reaction to **263** followed by enolisation at the α,β-unsaturated southern ketone (intermediate **263a**), and ring closure (**263b**) towards the northern ketone to give **264** after isomerisation of the eliminated intermediate **263c** which already contains the tigliane skeleton.

**Scheme 53:**
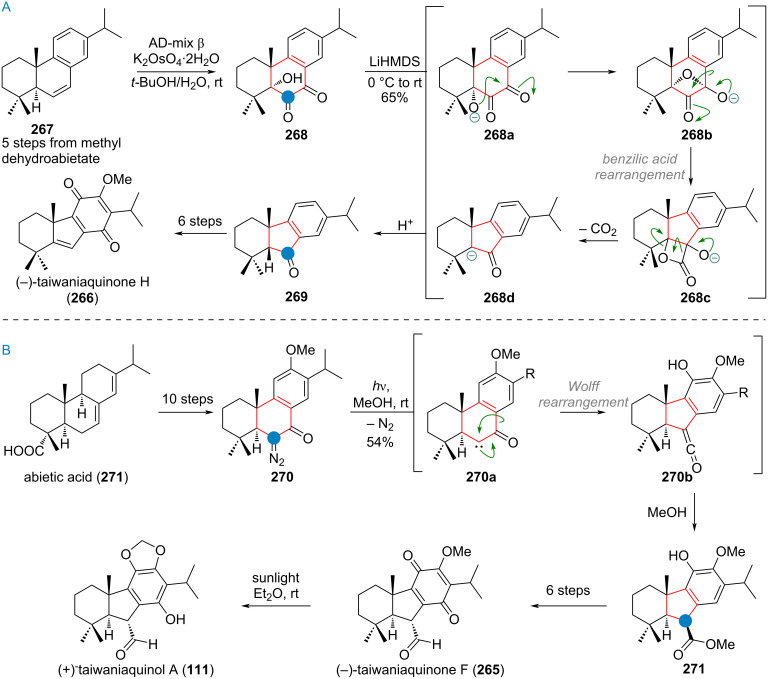
A: First generation synthesis of taiwaniaquinones by benzilic acid-type rearrangement of the B-ring; B: second generation approach towards taiwaniaquinols and taiwaniaquinones enabled by Wolff rearrangement.

The Gademann group reported two different approaches, both including a bioinspired 6→5 ring contraction for the synthesis of taiwaniaquinol A (**111**) and taiwaniaquinones F and H, **265** and **266**. In 2010, they synthesised the tricyclic precursor **267** (see Scheme 53A) from methyl dehydroabietate in 5 steps, followed by exhaustive oxidation at C-5, C-6, and C-7 by way of dihydroxylation, benzylic oxidation, and keto/enol tautomerism using AD-mix-β to diketone **268** [301].

The diketone **268** was then treated with a base to effect ring contraction via the following suggested mechanism: 4-membered acetal formation by attack of C-5 alkoxide (**268a**) to give **268b**, then 1,2-phenyl shift to generate the 4-membered lactone **268c**. Decarboxylation of this instable intermediate affords the anion **268d**, which was protonated to deliver **269**. The target natural compound taiwaniaquinone H (**266**) could be accessed in 6 further steps. In a second-generation synthesis (see Scheme 53B) the same group accessed the α-diazo ketone **270** in 10 steps from abundant and readily available abietic acid (**271**) [302]. A Wolff rearrangement was then carried out to effect ring contraction via carbene **270a** to the ketene **270b**, which was quenched with methanol to give the ester **271**. From here further 6 steps furnished taiwaniaquinone F (**265**) and a complex photolytic reaction (mechanism not secured but suspected to involve a 1,5-HAT of an excited quinone radical) led to taiwaniaquinol A (**111**) [303–309].

**Scheme 54:**
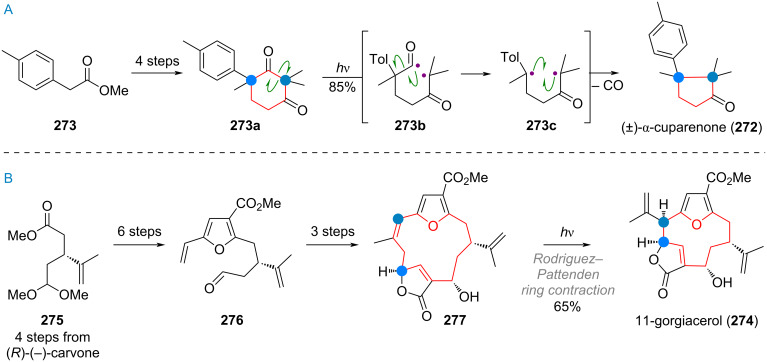
A: Norrish type 1 radical recombination leading to ring contraction en route to cuparenone (**272**): 13→11 ring contraction via photolysis of a furanocembrane in the synthesis of gorgiacerol (**274**) reported by Mulzer et al.

An isolated example for a ring contraction via CO expulsion in crystalline materials was reported by the Garcia-Garibay group for the synthesis of cuparenone (**272**) [310]. Methyl ester **273** was elaborated to the 6-membered diketone **273a**, which upon irradiation of the crystals selectively reacted, presumably via Norrish type I cleavage (**273b**), CO expulsion and recombination (**273c**) to give the target natural product **272**. The selectivity of this reaction was far superior in the crystalline form than in solution (see Scheme 54A). The Mulzer group, during their synthetic campaign into complex furanocembranoids, utilised a bioinspired 13→11 ring contraction to reach 11-gorgiacerol (**274**) [311]. Starting from dimethyl acetal **275**, the furan **276** was prepared in 6 steps (see Scheme 54B). Another three steps were required to elaborate the molecule and close the macrocycle to give **277**. From here, the Rodriguez–Pattenden ring contraction was applied [312–314]. Mechanistically, a *Z*→*E* isomerisation of the 7,8-double bond is invoked followed by diradical formation and opening of the macrocycle, and finally recombination gives the novel connectivity in **274**.

**Scheme 55:**
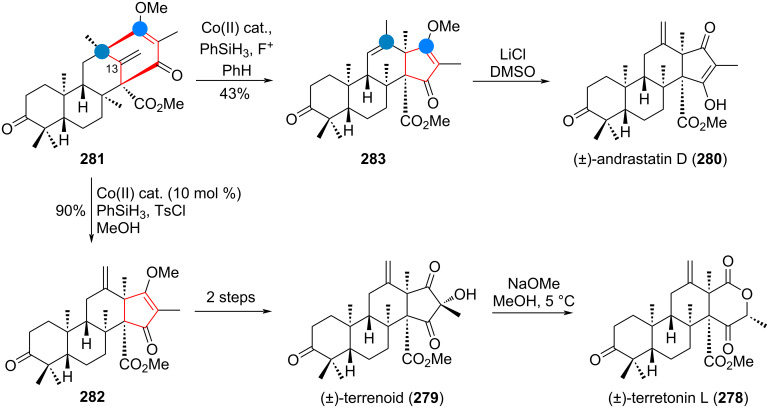
Ring contraction of a bridged D-ring system in the total synthesis of andrastatin D (**280**), terrenoid (**279**), and terretonin L (**278**).

Maimone, Newhouse and colleagues reported a synthesis of DMOA (= 3,5-dimethylorsellinic acid) meroterpenoids terretonin L (**278**), terrenoid (**279**), and andrastatin D (**280**) featuring intriguing ring contractions of the bridged tetracyclic system **281** [315]. A difference in reactivity was observed in the outcome of this radical reaction, depending on the precise conditions and additives used (see Scheme 55). Mechanistically, a C-13 centred radical is formed and attacks the α,β-unsaturated ketone of the bridged system. After cyclopropane rupture a C-12 radical is formed, which either (in the absence of F^+^) undergoes another HAT to give the exocyclic olefin **282** with only minor amounts of **283**. In turn, when a F^+^ source is added, almost perfect selectivity towards **283** is obtained, owing to likely oxidation of the C-12 radical to the cation and elimination. From here, the natural products **278**–**280** were accessed by demethylation, oxidation, and enol ether hydrolysis as well as retro-Claisen/transesterification.

**Scheme 56:**
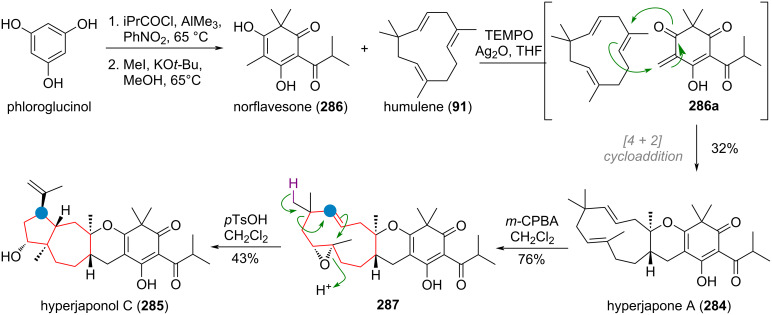
Biomimetic synthesis of hyperjapone A (**284**) and hyperjaponol C (**285**) by George et al.

During the biomimetic synthesis of hyperjapone A (**284**) and hyperjaponol C (**285**) by George and co-workers an elegant trans-annular cyclisation and concomitant ring contraction was showcased (see Scheme 56) [316]. The dearomatised diphenol norflavesone (**286**) was synthesised in two steps from phloroglucinol and then treated with humulene (**91**). Under oxidation with silver(I) oxide **286** reacted to the *o*-quinone methide **286a** ready for [4 + 2] cycloaddition with **91**. The product, hyperjapone A (**284**), was epoxidised to give **287** selectively and an ene-type transannular cyclisation followed by Wagner–Meerwein rearrangement and elimination furnished hyperjaponol C (**285**) upon treatment with a strong acid.

**Scheme 57:**
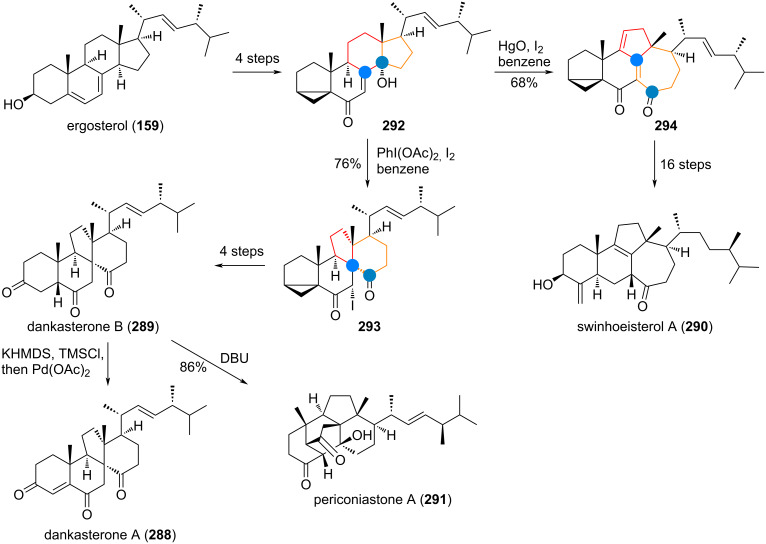
Heretsch’ synthesis of dankastarones A (**288**) and B (**289**), swinhoeisterol A (**290**), and periconiastone A (**291**) by bioinspired alkoxy radical triggered rearrangement.

Heretsch and co-workers demonstrated that radical reactions are equally powerful as the formation of charged intermediates to enact skeletal re-organisation (see Scheme 57). For the synthesis of dankasterones A (**288**) and B **289**, swinhoeisterol A (**290**), and periconiastone A (**291**) they prepared tertiary allylic alcohol **292** from ergosterol (**159**) in 4 steps [202]. Alkoxy radical formation under two different sets of conditions, triggered rearrangement (supposedly via the mechanisms discussed during the biosynthesis of these compounds, see Scheme 33) with capture of the radical by iodine giving **293** or directly to the 6/6/5/7 ring system of swinhoeisterol A (via **294**, reached in 16 additional steps). The α-iodoketone **293** was elaborated to dankasterone B (**289**) in 4 steps, and Saegusa oxidation delivered dankasterone A (**288**). Treatment of **289** with a strong base resulted in aldolisation towards the C-14 ketone to furnish the polycyclic compound periconiastone A (**291**).

**Scheme 58:**
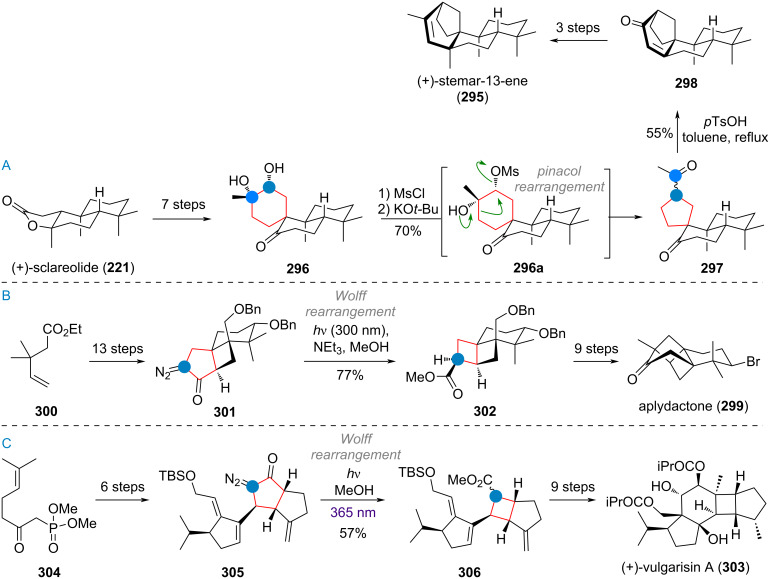
A: Zhang’s ring contraction during the synthesis of stemar-13-ene (**295**) by pinacol rearrangement; B: Ring contraction via Wolff rearrangement in Trauner’s synthesis of aplydactone (**299**); C: Wolff rearrangement for cyclobutene formation during Ding’s synthesis of vulgarisin A (**303**).

The pinacol rearrangement of a secondary/tertiary vicinal diol was utilised to great effect in Zhang’s total synthesis of stemar-13-ene (**295**) from sclareolide (**221,** see Scheme 58A) [317]. The 6/6-spirocyclic compound **296** was reached after 7 steps. Mesylation and treatment with strong base effected the desired bond migration (intermediate **296a**) towards 6/5-spirocycle **297**. Following an aldol reaction of the two carbonyls to give **298**, the synthesis was completed in three additional steps.

During Trauner’s synthesis of aplydactone (**299**) the Wolff rearrangement was once again used to synthesise one of the two cyclobutanes present in the molecule (see Scheme 58B) [318]. Starting from ester **300**, the diazoketone **301** was accessed in 13 steps and cleanly underwent the desired transformation in methanol to deliver the ester **302**. From here 9 additional steps were required to reach the target aplydactone (**299**). A similar 5→4 ring contraction via the Wolff rearrangement was reported by Ding and co-workers in their synthesis of vulgarisin A (**303**) [319]. The HWE reagent **304** was chosen as starting material and carried to bicyclic diazoketone **305** in just 6 steps. Carbene formation and 1,2-shift under irradiation in methanol delivered the methyl ester **306** as a mixture of diastereomers (see Scheme 58C). This was further modified to obtain vulgarisin A (**303**) in 9 additional steps.

**Scheme 59:**
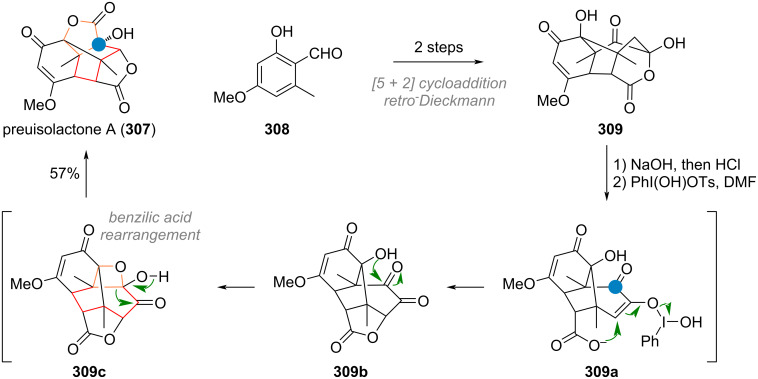
Trauner’s biomimetic synthesis of preuisolactone A (**307**) featuring a ring contraction via benzilic acid rearrangement.

During the biomimetic synthesis of preuisolactone A (**307**, see Scheme 59) by the group of Trauner, the hydroxybenzaldehyde **308** was converted into the bridged polycyclic framework of **309** in just 2 steps through dearomative dimerization via cycloaddition and retro-Dieckmann reaction, in analogy to the proposed biosynthesis [320–321]. Opening of the acetal (**309a**) and reaction with a hypervalent iodine reagent triggered oxidative lactonisation to triketone **309b**. Upon acetalisation (**309c**) of one of these two ketones a benzilic acid rearrangement effects the desired 1,2-alkyl migration, forming a 5-membered lactone ring and successfully contracting the carbocycle from 7→6 to deliver the product **307**.

**Scheme 60:**
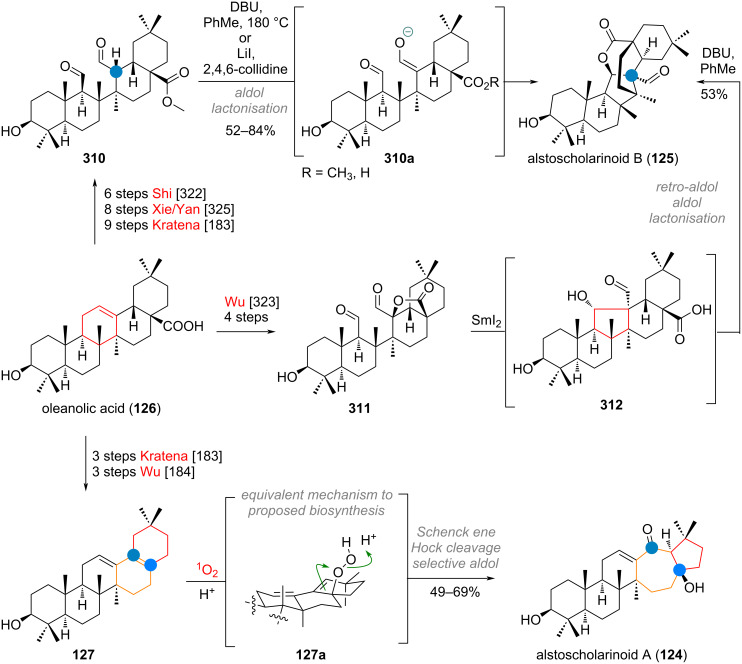
Bioinspired approaches for ring contraction/expansion reactions in the synthesis of alstoscholarinoids A (**124**) and B (**125**).

The two rearranged triterpenoids alstoscholarinoid A (**124**) and B **125**, with both contracted and expanded C, D, and E-rings were subject of several semisynthetic approaches (see Scheme 60), all starting from oleanolic acid (**126**) as the proposed biogenetic precursor (confer above, Scheme 30) [183–184,322–325]. For the C-ring 6→5-contracted compound **125**, three groups (Xie/Yan, Shi, and Kratena) independently developed approaches towards the crucial dialdehyde **310** as precursor for the intramolecular aldol addition and esterification to reach the target. This was achieved either by heating with a strong base (Shi, Kratena), leading to equilibration of aldol addition isomers and finally transesterification as an irreversible step, locking in place the configuration at C-11. Alternatively, the reaction could be realised by demethylation of the ester (Xie/Yan), with the carboxylate acting to deprotonate the α-proton of the C-12 aldehyde in **310a**. A clever solution to this problem was developed by Wu, who opted for an expedient synthesis of the dialdehyde **311** with a pending intramolecular lactone connecting to C-13. A reductive enolisation by treatment with SmI_2_ triggered aldol addition of the aldehydes, releasing the carboxylic acid in the process, giving the undesired C-11α-configured alcohol **312**. Retro-aldol and transesterification, also with DBU, lead to product **125** in just 6 steps. For alstoscholarinoid A (**124**), both the groups of Kratena and Wu reported independently that the previously reported oxidative cleavage of the tetrasubstituted olefin in aegiceradienol (**127**), leading to a diketone, was likely not responsible for the formation of this natural product. Instead, a biomimetic oxidation with singlet oxygen was found to give rise to tertiary hydroperoxide **127a** directly, which could undergo Hock rearrangement by addition of strong acid to finally reveal the enolised diketone, leading directly to aldol reaction and formation of the correct regioisomer alstoscholarinoid A (**124**).

**Scheme 61:**
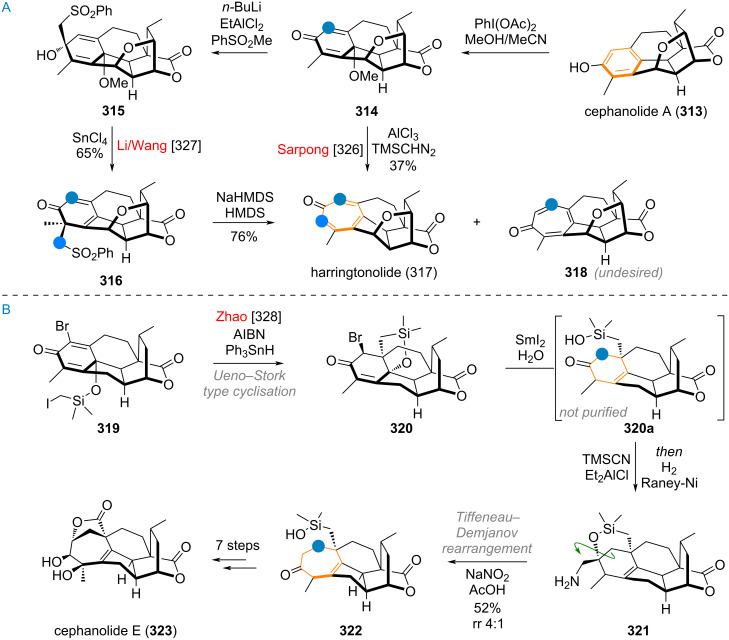
A: Sarpong and Li, Wang and co-workers’ ring expansion of cephanolide A (**313**) to reach harringtonolide (**317**); B: Zhao’s ring expansion via Tiffeneau–Demjanov rearrangement in the synthesis of cephanolide E (**323**).

As a final example, syntheses of C-19 diterpenoids from the genus *Cephalotaxus,* containing a seven-membered tropolone ring will be discussed. Recently, several groups have reported significant advances in ring expansion reactions of benzene rings at a late-stage during the synthesis of these molecules. Both the groups of Sarpong [326] and Li, Wang and co-workers [327] targeted harringtonolide (**317**) by ring expansion of the advanced intermediate **313** (see Scheme 61A). Dearomatisation towards the enone **314** allowed for nucleophilic attack of a methyl equivalent at the ketone to give **315**. Rearrangement with tin(IV) chloride effected 1,2-migration of the newly introduced group to give **316**. From here, harringtonolide (**317**) was reached by 1,2-addition of the sulfonyl carbon, cyclopropanation, and fragmentation to furnish the tropolone system. The groups of Sarpong and Zhao independently reported a direct ring expansion from intermediates such as **314** using diazomethane equivalents and Lewis acid. Apart from the desired natural product, the regioisomer **318** was produced in large amounts (55% for Sarpong’s approach). Zhao et al. [328] opted for substrates bearing additional halides on the rings, achieving an equal ratio of **317** and **318** by using Et_2_AlCl under optimised conditions. Additionally, Zhao’s synthesis also featured a more elaborate ring expansion utilised for constructing the complex diterpenoid cephanolide E (**323**, see Scheme 61B). The advanced, dearomatised intermediate **319** was cyclised via methylene radical addition towards the Michael acceptor, giving **320**. Reduction with samarium(II) iodide removed both the bromide and the silylated tertiary alcohol. The intermediate **320a** was not isolated, instead directly treated with TMSCN and Lewis acid, to effect cyanohydrin formation (not depicted) which was hydrogenated directly to provide **321**. This material is spring-loaded for ring expansion 1,2-shift towards a methyl cation. Upon diazotation the desired ring expansion took place smoothly to give **322**, which was still 7 steps from the target, cephanolide E (**323**).

## Conclusion

The remarkable structural diversity observed in terpenoids arises not only from the variety of cyclisation enzymes and oxidative follow-up functionalisation but also from intricate ring-size-modifying transformations, as comprehensively examined in this review. These skeletal rearrangements can proceed through diverse mechanistic pathways, including radical-mediated processes, cationic rearrangements, and oxidative cascades. Synthetic chemists have demonstrated remarkable ingenuity in recreating these complex transformations in the laboratory. These biomimetic syntheses not only provide access to structurally complex natural products in an efficient manner but also serve as powerful tools for validating proposed biosynthetic hypotheses. The interconversion between related terpenoid families through such skeletal reshuffling underscores the evolutionary efficiency of these chemical transformations in generating structural diversity in nature and chemical synthesis. Looking forward, the continued exploration of bioinspired ring contraction and expansion strategies promises to advance both fundamental understanding and practical applications in natural product synthesis. As enzymatic mechanisms become better characterised and synthetic methodologies continue to evolve, we anticipate the emergence of increasingly sophisticated approaches of skeletal editing that will further serve to close the gap between biosynthetic ingenuity and our own synthetic capability.

## Data Availability

Data sharing is not applicable as no new data was generated or analyzed in this study.
